# Association of peripheral blood DNA methylation level with Alzheimer’s disease progression

**DOI:** 10.1186/s13148-021-01179-2

**Published:** 2021-10-15

**Authors:** Qingqin S. Li, Aparna Vasanthakumar, Justin W. Davis, Kenneth B. Idler, Kwangsik Nho, Jeffrey F. Waring, Andrew J. Saykin, for the Alzheimer’s Disease Neuroimaging Initiative (ADNI)

**Affiliations:** 1 Neuroscience, Janssen Research and Development, LLC, 1125 Trenton-Harbourton Road, Titusville, NJ 08560 USA; 2 Genomics Research Center, AbbVie, North Chicago, IL USA; 3 Indiana Alzheimer’s Disease Research Center, Department of Radiology and Imaging Sciences, Indiana University School of Medicine, Indianapolis, IN USA

**Keywords:** Epigenetics, EWAS, DMP, DMR, Alzheimer’s disease

## Abstract

**Background:**

Identifying biomarkers associated with Alzheimer’s disease (AD) progression may enable patient enrichment and improve clinical trial designs. Epigenome-wide association studies have revealed correlations between DNA methylation at cytosine-phosphate-guanine (CpG) sites and AD pathology and diagnosis. Here, we report relationships between peripheral blood DNA methylation profiles measured using Infinium® MethylationEPIC BeadChip and AD progression in participants from the Alzheimer’s Disease Neuroimaging Initiative (ADNI) cohort.

**Results:**

The rate of cognitive decline from initial DNA sampling visit to subsequent visits was estimated by the slopes of the modified Preclinical Alzheimer Cognitive Composite (mPACC; mPACC_digit_ and mPACC_trailsB_) and Clinical Dementia Rating Scale Sum of Boxes (CDR-SB) plots using robust linear regression in cognitively normal (CN) participants and patients with mild cognitive impairment (MCI), respectively. In addition, diagnosis conversion status was assessed using a dichotomized endpoint. Two CpG sites were significantly associated with the slope of mPACC in CN participants (*P* < 5.79 × 10^−8^ [Bonferroni correction threshold]); cg00386386 was associated with the slope of mPACC_digit_, and cg09422696 annotated to *RP11-661A12.5* was associated with the slope of CDR-SB. No significant CpG sites associated with diagnosis conversion status were identified. Genes involved in cognition and learning were enriched. A total of 19, 13, and 5 differentially methylated regions (DMRs) associated with the slopes of mPACC_trailsB_, mPACC_digit_, and CDR-SB, respectively, were identified by both comb-p and DMRcate algorithms; these included DMRs annotated to *HOXA4*. Furthermore, 5 and 19 DMRs were associated with conversion status in CN and MCI participants, respectively. The most significant DMR was annotated to the AD-associated gene *PM20D1* (chr1: 205,818,956 to 205,820,014 [13 probes], Sidak-corrected *P* = 7.74 × 10^−24^), which was associated with both the slope of CDR-SB and the MCI conversion status.

**Conclusion:**

Candidate CpG sites and regions in peripheral blood were identified as associated with the rate of cognitive decline in participants in the ADNI cohort. While we did not identify a single CpG site with sufficient clinical utility to be used by itself due to the observed effect size, a biosignature composed of DNA methylation changes may have utility as a prognostic biomarker for AD progression.

**Supplementary Information:**

The online version contains supplementary material available at 10.1186/s13148-021-01179-2.

## Background

Nearly all (99.6%) clinical trials of Alzheimer’s disease (AD) that were registered at ClinicalTrials.gov during 2002 to 2012 failed, likely because of inadequate understanding of disease pathways and drug targets and difficulty in identifying patients at early stages of the disease [1]. A total of 2621 trials for AD have been completed, yet the disease remains incurable [2]. Over the years, the United States Food and Drug Administration (FDA) has approved a limited number of drugs that provide symptomatic relief [3]. Although no curative treatments currently exist for AD, several disease-modifying therapies that aim to slow disease progression, especially during early stage, are under investigation [4, 5]. As of January 5, 2021, 126 investigative agents were in clinical trials for AD; most of them targeting the biological processes underlying AD to modify the disease [6]. Several biomarkers that may be correlated with disease progression and prognosis are being pursued. A new drug that reduces beta-amyloid (Aβ) plaques and may delay disease progression in patients with AD recently received an accelerated FDA approval [7].

The Alzheimer’s Disease Neuroimaging Initiative (ADNI) is an important data source available to the scientific community that greatly contributes to the understanding of AD progression [8]. An analysis of cerebrospinal fluid (CSF) samples from 287 participants in the ADNI database over 3 to 6 years identified a 16-peptide signature that could not only differentiate between patients with AD and cognitively normal (CN) participants but could also predict progression from mild cognitive impairment (MCI) to AD better than the traditional Aβ and tau biomarkers [9]. Similarly, a 24-month single-center imaging study showed that deposition of Aβ and cortical thickness are more sensitive biomarkers of disease progression in early AD compared with neuronal dysfunction [10]. Another recent study of patients from both Japanese and North American ADNI databases, which defined disease progression based on the change in Clinical Dementia Rating Scale Sum of Boxes (CDR-SB) scores (≥ 1, progression; < 1, stable), identified several prognostic factors in early AD: baseline tau protein levels in CSF and scores from Mini-Mental State Examination (MMSE), Functional Activities Questionnaire, and 13-item Alzheimer’s Disease Assessment Scale-cognition subscale [11].

Neuroinflammation and activated microglia are also considered to play key roles in AD progression by interacting with Aβ pathways [12], which may offer additional biomarkers of AD progression. A recent study showed a correlation between baseline levels of CSF cytokine CCL2 and the rate of cognitive decline in patients with MCI who have CSF biomarkers consistent with AD [13]. Furthermore, machine learning algorithms are being used to predict disease progression. A neural network model based on data from 1737 participants in the ADNI database was effective in predicting AD progression in a separate set of 110 participants who were CN or had MCI at baseline [14]. Other machine learning-based models have employed neuroimaging and noninvasive methods using blood biomarkers to predict AD progression [15, 16].

Despite these promising leads, additional biomarkers such as DNA methylation status may shed light on disease progression and potentially shorten the duration of clinical trials of AD. DNA methylation in peripheral blood has been used as a diagnostic biomarker in other diseases [17–19]. Although studies evaluating epigenetic markers in peripheral blood have shown differential DNA methylation in patients with MCI or AD compared with CN participants, suggesting that a DNA methylation-based biosignature could potentially serve as a surrogate of AD diagnosis [20–23], these studies did not use longitudinal clinical data to assess whether peripheral DNA methylation might be associated with disease progression and prognosis. Peripheral DNA methylation was recently shown to be associated with normal brain aging and cognitive decline (e.g., cognitively impaired [Montreal Cognitive Assessment or MoCA < 26]) vs. cognitively normal [MoCA > 26]) [24]. DNA methylation in blood was previously studied in nonconverters versus converters to AD using Infinium® HumanMethylation450K BeadChip [25]; however, to our knowledge, it has not been profiled as a biomarker of AD progression and prognosis using the higher density Infinium® MethylationEPIC BeadChip.

This study aimed to investigate the relationship between baseline peripheral DNA methylation level and disease progression. The earliest available DNA sample from a participant (baseline) was analyzed to assess whether the epigenetic profile obtained at a single clinical visit is associated with the disease trajectory. Cognitive changes in patients across different stages of the disease continuum are captured using different scales, each presumably most sensitive to change in cognitive function for a specific disease stage. For example, the Alzheimer Disease Cooperative Study-Preclinical Alzheimer Cognitive Composite (PACC) was designed as the primary outcome measure of cognitive decline in patients at the asymptomatic phase of AD [26]. Using the PACC score at 24 months to measure cognitive decline in study participants with preclinical AD, Donohue et al. previously showed that Aβ-positive participants had significantly more decline than Aβ-negative participants [26]. Accordingly, the PACC, adapted for the available tests in ADNI [27], was used in the present study to assess cognitive decline in participants who were CN at baseline. The CDR-SB scale, which provides more detailed staging information compared with CDR global score for patients with MCI [28], was used to assess cognitive decline in patients with MCI at baseline to optimize the sensitivity to change in this population [29, 30]. Lower PACC scores and higher CDR-SB scores represent poorer cognitive performance. In addition, dichotomized endpoints were used to assess conversion status in both CN participants and patients with MCI.

## Results

### Disease progression

The association of DNA methylation with disease progression was analyzed in 2 diagnosis groups: CN and MCI. Among 202 participants in the CN group, 56 (27.7%) converted from CN at baseline to MCI (converters) and 146 (72.3%) did not (nonconverters), at up to 8 years since DNA sampling. The mean (SD) follow-up period since DNA sampling in the CN group was 54.1 (21.5) months for converters and 61.2 (19.4) months for nonconverters. Among 317 patients in the MCI group, 115 (36.3%) converted from MCI to AD and 202 (63.7%) were nonconverters. The mean (SD) follow-up time since DNA sampling in the MCI group was 43.6 (20.9) months for converters and 57.2 (18.1) months for nonconverters.

### Differentially methylated positions in peripheral blood

**Fig. 1:**
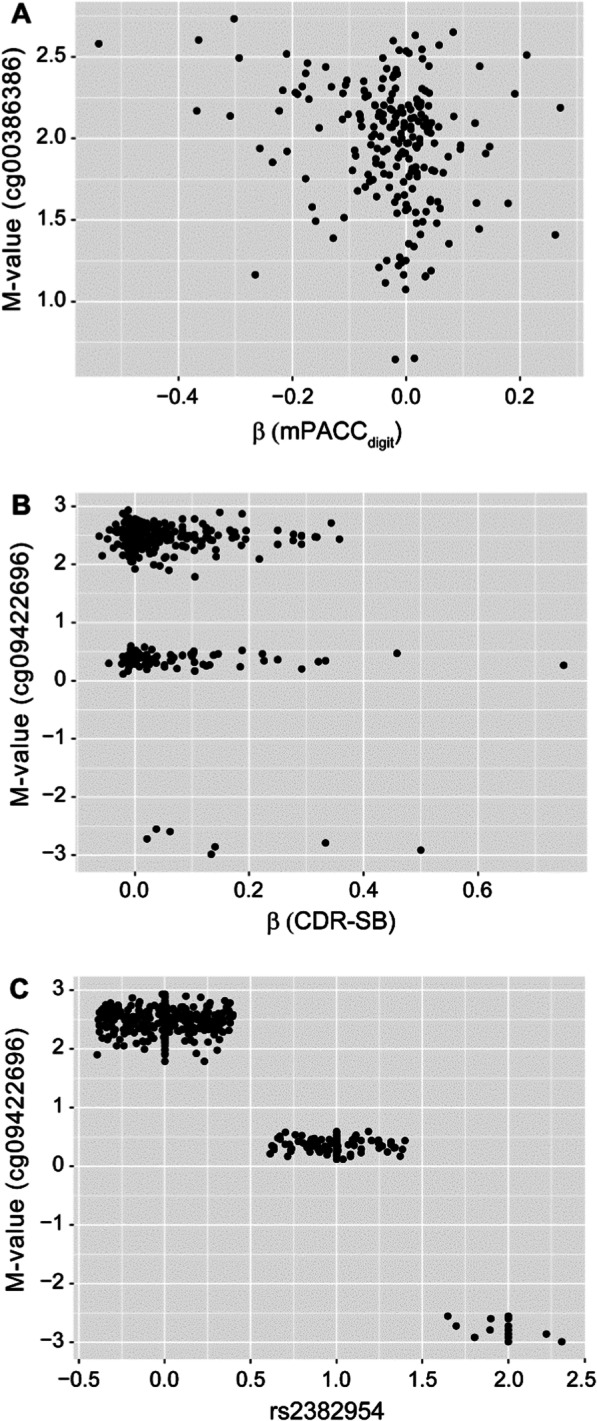
Association of CpG sites with rate of cognitive decline. **A** cg00386386 was associated with the slope of mPACC_digit_ in CN participants. **B** cg09422696 was associated with the slope of CDR-SB in patients with MCI. **C** rs2382954–cg09422696 mQTL. Note that jitter was introduced for the *x*-axis. *CDR-SB* Clinical Dementia Rating Scale Sum of Boxes, *CN* cognitively normal, *MCI* mild cognitive impairment, *mPACC*_*digit*_ modified Preclinical Alzheimer Cognitive Composite that used Digit Symbol Substitution Test, *mQTL* methylation quantitative trait locus

**Fig. 2:**
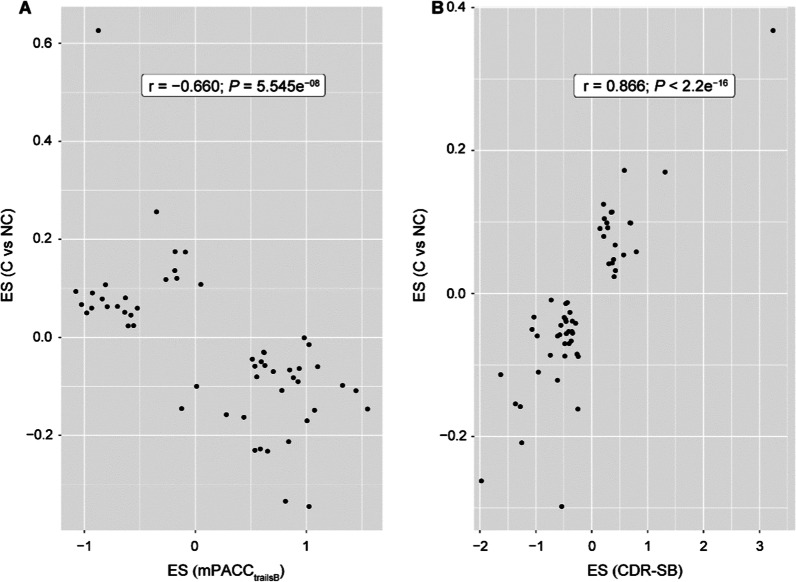
The correlation of the effect size from DMP analysis for the slope of mPACC_trailsB_ conversion status in CN participants (**A**) and the slope of CDR-SB vs. conversion status in patients with MCI (**B**). Only the probes with *P* < 1 × 10^–5^ in either analysis are plotted. *CDR-SB* Clinical Dementia Rating Scale Sum of Boxes, *CN* cognitively normal, *DMP* differentially methylated positions, *MCI* mild cognitive impairment, *mPACC*_*trailsB*_ modified Preclinical Alzheimer Cognitive Composite that used Trail-Making Test Part B

Two Cytosine-phosphate-guanine (CpG) sites were associated with the rate of cognitive decline in CN participants and patients with MCI at a level surpassing Bonferroni correction threshold of *P* = 5.79 × 10^–8^ (Table 1). After BACON correction [31], all lambda values were < 1.04 (Additional file 1: Fig. 1 [Q-Q plots]; Fig. 2 [Manhattan plots]). In CN participants, cg00386386 was associated with the rate of cognitive decline as measured by the slope of modified PACC (mPACC) that used mPACC_digit_ (*P* = 6.50 × 10^−9^) (Table 1; Fig. 1A). In patients with MCI, cg09422696 annotated to *RP11-661A12.5* was associated with the rate of cognitive decline as measured by the slope of CDR-SB (*P* = 1.17 × 10^−8^) (Fig. 1B, Table 1). Single nucleotide polymorphism (SNP) rs2382954 was correlated with DNA methylation level at cg09422696 (r =  − 0.98, *P* < 2.2e^−16^) and hence a methylation quantitative trait locus (mQTL) in *cis* (Fig. 1C), as reported previously [32]. The SNP rs2382954 was also associated with the slope of CDR-SB (*P* = 8.12 × 10^−5^). When adjusted for rs2382954, the association between cg09422696 methylation level and the slope of CDR-SB failed to meet the significance threshold (*P* > 0.05), suggesting that the association was driven solely by rs2382954. A list of *P* values suggestive of associations of other CpG sites with the rate of cognitive decline (*P* < 1 × 10^−5^) is available in Additional file 2: Table 1. No CpG probe was significantly associated with disease conversion status (ie, CN to MCI or MCI to AD) (Additional file 1: Figs. 1C, E, 2C, and E) passing Bonferroni correction threshold.

**Table 1:** List of DMPs with BACON-corrected *P* values less than Bonferroni correction threshold^a^

Name	Chr	Pos	GencodeCompV12	Beta	Ave *M*-value	*t*	Before BACON correction		After BACON correction		Phenotype
							*P* value	Adjusted *P* value	*P* value	Adjusted *P* value	
*Without APOE4 covariate in the model*											
cg00386386	Chr9	136,216,149	MED22;RPL7A;SNORD24;SNORD36B;SNORD36A	− 0.97	1.97	− 6.14	4.12E^−09^	3.56E^−03^	6.50E^−09^	5.61E^−03^	mPACC_digit_
cg09422696	Chr8	144,626,190	RP11-661A12.5	− 1.63	1.89	− 5.27	2.45E^−07^	2.12E^−01^	1.17E^−08^	1.01E^−02^	CDR-SB
*With APOE4 covariate in the model*											
cg00386386	Chr9	136,216,149	MED22;RPL7A;SNORD24;SNORD36B;SNORD36A	− 0.97	1.97	− 6.17	3.59E^−09^	3.10E^−03^	6.34E^−09^	5.48E^−03^	mPACC_digit_
cg09422696	Chr8	144,626,190	RP11-661A12.5	− 1.65	1.89	− 5.38	1.41E^−07^	1.22E^−01^	9.87E^−09^	8.53E^−03^	CDR-SB

*CDR-SB* Clinical Dementia Rating Scale Sum of Boxes, *chr* chromosome, *GencodeCompV12* GENCODE Comprehensive database version 12 containing all transcripts at protein-coding loci, *mPACC*_*digit*_ modified Preclinical Alzheimer Cognitive Composite that used Digit Symbol Substitution Test, *Pos* position in reference to genome build hg19

^a^*P* = 5.79 × 10^−8^

Using all baseline study samples from the ADNI cohort, mPACC_digit_ was negatively correlated with CDR-SB (*r* =  − 0.787, *P* < 2.2e^−16^) and highly correlated with mPACC_trailsB_ (*r* = 0.980; *P* < 2.2e^−16^) (Additional file 1: Fig. 3). For participants who were CN at baseline, the correlation between mPACC_digit_ and CDR-SB was reduced (*r* =  − 0.128; *P* = 0.004), and the dynamic range for mPACC_digit_ was wider than that for CDR-SB (0–1) (Additional file 1: Fig. 4A), suggesting that mPACC_digit_ measures the cognitive change in an asymptomatic population. Despite the significant but attenuated correlation between mPACC_digit_ and CDR-SB among baseline measurements from participants who were MCI at baseline (*r* =  − 0.324, *P* < 2.2e^−16^, Additional file 1: Fig. 4B), the range of mPACC_digit_ did not seem to vary much for participants with CDR-SB ranging from 0 to 5.5, supporting the choice of using CDR-SB to capture the change in cognitive function among patients who were MCI at baseline. Additional file 1: Fig. 4C shows correlation between mPACC_digit_ and CDR-SB for participants who were either CN or MCI at baseline (*r* =  − 0.637; *P* < 2.2e^−16^).

**Fig. 3:**
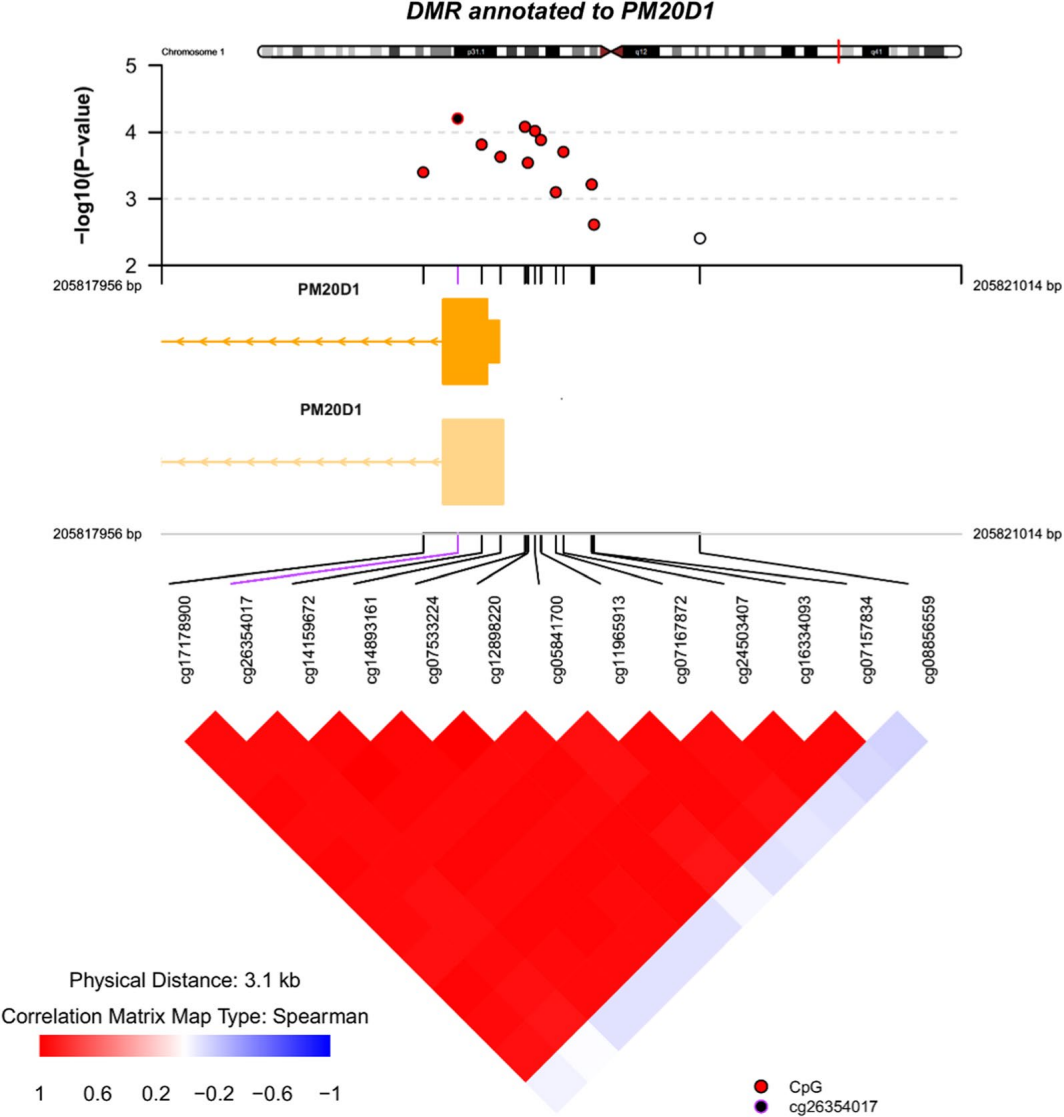
*PM20D1* DMR association with the conversion status in patients with MCI (e.g., converters vs. nonconverters). Top panel: individual CpG association *P*-values; middle panel: gene structure; bottom panel: pairwise correlation between CpG sites in this DMR. *CpG* cytosine-phosphate-guanine, *DMR* differentially methylated region, *MCI* mild cognitive impairment

**Fig. 4:**
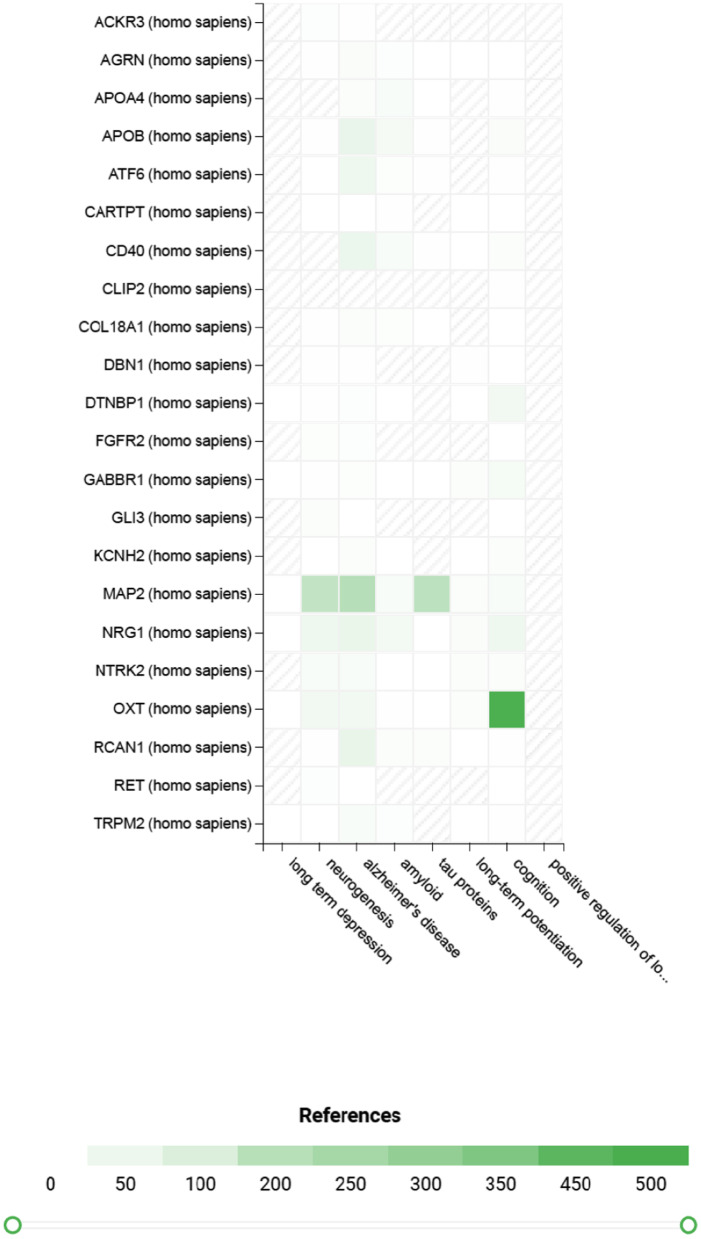
Heatmap of co-occurrence in PubMed abstracts among genes annotated by DMRs across all endpoints and key concepts enriched in other analysis or biological processes important in AD. Hashed square means no reference was identified by Euretos AI Platform. Genes with 5 references associated with cognition, neurogenesis, amyloid, tau proteins are shown

The effect sizes for the association between the slope of mPACC_trailsB_ and methylation level of CpG probes were correlated with the effect sizes for mPACC_digit_ (*r* = 0.997, *P* < 2.2e^−16^ for probes with *P*_DMP_ < 1 × 10^−5^ in either analysis). The effect sizes for the slope of mPACC_trailsB_ were negatively correlated with the effect sizes for the conversion status in CN participants (*r* =  − 0.660, *P* = 5.545e^−8^ for probes with *P* < 1 × 10^−5^ in either analysis, Fig. 2A) but not correlated with the effect sizes for the slope of CDR-SB (*r* = 0.020, *P* = 0.860) or the effect sizes for the conversion status in patients with MCI (*r* =  − 0.024, *P* = 0.868) for the probes with *P* < 1 × 10^−5^ in either analysis. In contrast, the effect sizes for the slope of CDR-SB were correlated with those for conversion status in patients with MCI (*r* = 0.866, *P* < 2.2e^−16^ for the probes with *P* < 1 × 10^−5^ in either analysis; Fig. 2B), suggesting that the effect sizes from the continuous endpoints are concordant with those from the paired dichotomized endpoints, but the effect sizes in the two populations (CN vs. MCI at baseline) are quite far apart.

Gene set enrichment analysis (GSEA) using methylGSA revealed enrichment of mitogen-activated protein kinase signaling pathway (*P* = 0.002, adjusted *P* = 0.05) and Fc epsilon RI signaling pathway (*P* = 0.01, adjusted *P* = 0.14) for the rate of cognitive decline as measured by the slope of mPACC_trailsB_ (Additional file 2: Table 2A). Overrepresentation analysis of CpG probes with nominal *P* < 0.01 revealed a common theme of enrichment of genes involved in neurogenesis for the rate of cognitive decline as measured by the slopes of mPACC_trailsB_ (Additional file 2: Table 2A), mPACC_digit_ (Additional file 2: Table 2B), CDR-SB (*P* = 7.00 × 10^−6^, FDR adjusted *P* = 0.006, Additional file 2: Table 2C), and for conversion status in the CN participants (Additional file 2: Table 2D). Gene sets involved in cognition were also nominally enriched for the rate of cognitive decline as measured by the slope of mPACC_trailsB_ (*P* = 0.0007, adjusted *P* = 0.07, Additional file 2: Table 2A) and conversion status in patients with MCI (*P* = 0.0001, Additional file 2: Table 2E).

We compared our results with the analyses of follow-up samples from 2 studies—AddNeuroMed (comparing MCI to AD converters with nonconverters) and Middle-Aged Danish Twin (MADT) studies—which evaluated the relationship between epigenetic profiles and cognitive changes over 10 years [33, 34]. The effect size from the top 1000 DMPs from the AddNeuroMed study [33] was weakly correlated with the effect size from the MCI to AD conversion status in this study (r = 0.1, *P* = 0.002, Additional file 1: Fig. 5). There are 22 CpG sites associated with disease progression with consistent directionality and nominal *P* < 0.05 between this study and AddNeuroMed study (Additional file 2: Table 3), including cg20152430 (*P* = 0.003 in AddNeuroMed; *P* = 0.003 in this study) that annotated to *HOXB3* and cg12559197 (*P* = 0.001 in the AddNeuroMed study; *P* = 0.0008 in this study) that annotated to *PDE8B.* In the MADT study [34], only 12 probes were reported to be associated with cognitive change over 10 years, among which cg27630540 annotated to *KIAA1530* (*β* =  − 0.01, *P* = 8.25 × 10^−6^ in the MADT study) trended in the same direction (*β* =  − 0.39, *P* = 0.06) as the slope of mPACC_trailsB_ analysis in this study, and cg13630845 annotated to *SLC35E1* (*β* =  − 0.0247, *P* = 7.73 × 10^−6^ in the MADT study) trended in the same direction as observed in the CN to MCI converter versus nonconverter analysis (*β* = 0.04, *P* = 0.1).

There was no correlation at the CpG level between the effect sizes for the slope of either mPACC_trailsB_ or CDR-SB and the effect size for the 220 CpG sites associated with Braak stage across cortex in the brain previously identified in an epigenome-wide association meta-analysis study (EWAS) [35] (Additional file 1: Figs. 6A, B). Similarly, there was no correlation between the effect sizes for the clinical diagnosis (AD vs. CN) in blood published previously [20] and the effect size for the 220 CpG sites across cortex in the EWAS meta-analysis (Additional file 1: Fig. 6C), suggesting a generally low concordance between peripheral blood and brain, although the compared phenotypes are not identical, and the blood and brain data were not from the same individuals. In addition, there was no correlation at the CpG level between the effect sizes for the slope of either mPACC_trailsB_ or CDR-SB and the effect size for AD diagnosis in blood from the same ADNI cohort [20] (Additional file 1: Fig. 7A and B).

### Differentially methylated region (DMR) analysis

DMR analysis enabled identification of regions in the genome consisting of ≥ 3 probes. Overall, comb-p algorithm identified 70 and 76 DMRs to be significantly associated with the rate of cognitive decline as measured by the slopes of mPACC_trailsB_ and mPACC_digit_, respectively, in participants who were CN at baseline (Additional file 2: Tables 4A and B). Among these, 47 DMRs were common between the two endpoints, including a DMR annotated to *HOXA4* (chr7: 27,170,241 to 27,171,051 [14 probes], Sidak-corrected *P* = 3.26 × 10^–6^) associated with the slope of mPACC_trailsB_ (Additional file 1: Fig. 8A) and an overlapping DMR annotated to *HOXA4* (chr7: 27,169,957 to 27,171,154 [16 probes], Sidak-corrected *P* = 1.24 × 10^–10^) associated with the slope of mPACC_digit_ (Additional file 1: Fig. 8B). In blood, the increased levels of methylation or probes in the DMR annotated to *HOXA4* were associated with slower rate of cognitive decline (Additional file 1: Fig. 8A). In addition, 5 DMRs annotated to the protocadherin gamma gene cluster were also identified as associated with the slope of mPACC_digit_, including DMRs annotated to *PCDHGA4* (chr5: 140,734,648 to 140,735,027 [4 probes], Sidak-corrected *P* = 0.002), *PCDHGA10* (chr5: 140,794,664 to 140,794,993 [3 probes], Sidak-corrected *P* = 0.007), *PCDHGB7* (chr5: 140,796,045 to 140,796,312 [3 probes], Sidak-corrected *P* = 0.008), *PCDHGA11* (chr5: 140,802,432 to 140,802,831 [4 probes], Sidak-corrected *P* = 0.002), and *PCDHGA12* (chr5: 140,810,051 to 140,810,433 [9 probes], Sidak-corrected *P* = 1.75 × 10^–5^). DMRcate detected fewer DMRs (24 and 16 for mPACC_trailsB_ and mPACC_digit_, respectively), among which 19 and 13 were also detected by comb-p. The DMRs annotated to *HOXA4* and *PCDHGA12* were detected by both methods.

A total of 102 and 5 DMRs, detected by comb-p and DMRcate, respectively (all 5 detected by DMRcate were also detected by comb-p), were significantly associated with the rate of cognitive decline as measured by slope of CDR-SB in patients who had MCI at baseline (Sidak-corrected *P* < 0.05, Additional file 2: Table 4C). This included a DMR annotated to *HOXB6* (detected by comb-p only, chr17: 46,681,111–46,681,550 [7 probes], Sidak-corrected *P* = 0.0002) and a DMR annotated to *HOXB9* (detected by comb-p only, chr17: 46,698,598–46,699,155 [7 probes], Sidak-corrected *P* = 0.002); however, one DMR was hypermethylated and the other was hypomethylated (Additional file 1: Figs. 8C and D).

For the dichotomized conversion status association, 75 and 123 DMRs were identified by comb-p in study participants who were CN or had MCI at baseline, respectively (Additional file 2: Tables 4D and 4E), replicating two previously reported DMRs (*KCNAB3* and *GABBR1*) associated with MCI conversion status from the AddNeuroMed study [33]. For the probe annotated to *GABBR1* among the top 1000 DMPs associated with MCI to AD conversion status in the AddNeuroMed study (cg06512249, *β* =  − 0.03, *P* = 0.003), the direction was consistent with multiple probes in the same region from this study (cg03316098, *β* =  − 0.05, *P* = 0.01; cg10234998, *β* =  − 0.10, *P* = 0.005; cg12061917, *β* =  − 0.07, *P* = 0.001; cg21481950, *β* =  − 0.06 *P* = 0.007) even though cg06512249 did not reach the nominally significant threshold in this study (*P* > 0.05) (Additional file 1: Fig. 8E). The probe from the *KCNAB3* was not among the top 1000 DMPs associated with MCI to AD conversion status in the AddNeuroMed study, so the directionality could not be confirmed in this study. The most significant DMR identified in this study that was associated with conversion status in patients with MCI was annotated to *PM20D1* (chr1: 205,818,956 to 205,820,014 [13 probes], Sidak-corrected *P* = 7.74 × 10^−24^) (Fig. 3). A similar DMR annotated to *PM20D1* (chr1: 205,818,956 to 205,819,609 [12 probes], Sidak-corrected *P* = 7.00 × 10^−5^) was also associated with the rate of cognitive decline as measured by the slope of CDR-SB in patients with MCI. In addition, a DMR annotated to *DUSP22* (chr6: 291,634 to 292,823 [10 probes], Sidak-corrected *P* = 8.09 × 10^−14^, Additional file 1: Fig. 8F) was associated with conversion status in CN participants. DMRcate identified fewer DMRs (6 and 21 in study participants who were CN or had MCI at baseline, respectively), among which 5 and 19, respectively, overlapped with those identified by comb-p. The DMRs annotated to *PM20D1* and *DUSP22* were detected by both DMR methods.

Although the DMRs annotated to *OXT* and *LDLRAD4* associated with conversion status to AD in this study and the German Study on Aging, Cognition and Dementia in Primary Care Patients (AgeCoDe) [25], the probes in this study were hypomethylated among converters in contrast to the reported hypermethylation in blood in the AgeCoDe cohort study. Another DMR annotated to *PRRT1* that associated with conversion status to AD in the AgeCoDe study, was also associated with both the slope of mPACC_trailsB_ (detected by both comb-p and DMRcate) and CDR-SB in this study but in opposite direction.

Multiple other DMRs were detected: *ZFP57* (identified by both comb-p and DMRcate); *LMTK3* (Additional file 1: Fig. 8G) and *FBXO44* (identified by comb-p only) for the rate of cognitive decline as measured by the slope of CDR-SB in patients with MCI (Additional file 2: Table 4C); *LOC105375131* and *GAL3ST2* (both identified by comb-p alone) for conversion status in CN participants (Additional file 2: Table 4D); and *FGFR2* (identified by both comb-p and DMRcate) for conversion status in patients with MCI (Additional file 2: Table 4E).

Furthermore, DMRs annotated to *MORN4*, *AIRE, LY6G5C*, and *PRRT1* were identified for both rates of cognitive decline as measured by the slopes of mPACC_digit_/mPACC_trailsB_ and CDR-SB. In addition, DMRs annotated to *FOXK1*, *ACY3*, and *ZBED9* were identified for conversion status in both subjects with CN and MCI at baseline. With the exception of the DMR annotated to *ACY3* that was detected by both comb-p and DMRcate, the rest of DMRs were detected by comb-p alone. The overlap between DMRs association in the CN and MCI populations appears to be small, consistent with the low correlation of effect size in DMP analysis between the two populations. In contrast, there were 47 overlapped DMRs between the rate of cognitive endpoints defined by the slope of mPACC_digit_ versus mPACC_trailsB._

Genes involved in neuronal postsynaptic density were enriched among DMRs associated with the rate of cognitive decline as measured by mPACC_trailsB_, and this enrichment was driven by DMRs annotated to *BNIP3*, *NTRK2*, and *BAIAP2* (Additional file 2: Table 5A). Similarly, genes involved in regulation of postsynaptic density assembly or organization were enriched among DMRs associated with conversion status in CN participants (Additional file 2: Table 5B). Notably, genes encoding neural cadherin-like cell adhesion proteins are highly enriched among the DMRs associated with the slope of mPACC_digit_ (Additional file 2: Table 5C). Additional ontology terms that were enriched are shown in Additional file 2: Tables 5D and E. Furthermore, genes with literature evidence linking to neurogenesis, cognition, amyloid, and tau pathology are shown in Fig. 4 and Additional file 2: Table 6.

## Discussion

We assessed the association between disease progression and DNA methylation in CN participants and patients with MCI. Approximately 30% of CN participants converted to MCI and approximately 36% of patients with MCI converted to AD in a mean follow-up period of 54.1 and 43.6 months, respectively. Two differentially methylated positions (DMPs) were significantly associated with cognitive decline at a level surpassing Bonferroni correction threshold: cg00386386 was associated with the slope of mPACC_digit_ in CN participants, and cg09422696 was associated with the slope of CDR-SB in patients with MCI. When adjusted for rs2382954, the association between cg09422696 methylation level and the slope of CDR-SB was no longer statistically significant, suggesting that the association was driven solely by rs2382954. No CpG sites were identified that were significantly associated with diagnosis conversion status.

In this study, we found several DMRs previously implicated in AD, including those annotated to *HOXA4*, *HOXB6/HOXB9* that were associated with the rate of cognitive decline as measured by the slopes of mPACC_trailsB_/mPACC_digit_, and CDR-SB, respectively; and those annotated to *DUSP22* and *PM20D1* that were associated with conversion status in CN and MCI participants, respectively. The DMRs annotated to *HOXA4* and *HOXB6*/*HOXB9* gene clusters have been previously shown to be associated with tau pathology and/or neurogenesis [33, 35–42]. The decreased levels of methylation for probes in the DMR annotated to *HOXA4* were associated with faster rate of cognitive decline; in contrast, hypermethylation was reported for probes annotated to the *HOXA* gene cluster in the brain in patients with AD [24, 35, 40–42]. A cell-type specific EWAS suggested that the hypermethylation was derived from neuronal cells [43]. In the present study, the hypermethylation of probes in the DMRs annotated to *HOXB6* was associated with faster rate of cognitive decline, whereas the hypermethylation of probes in the DMRs annotated to *HOXB9* was associated with slower rate of cognitive decline. The hypermethylation in the *HOXB* gene cluster and *HOXB6* gene were previously reported in brain and blood, respectively [33, 35, 41]; in contrast, expression of *HOXB9* was reported to be increased in the brain tissue from patients with Huntington disease [44]. The effect sizes for the association between CpG and the slope of neither mPACC_trailsB_ nor CDR-SB were correlated with the effect sizes for the 220 CpG sites across cortex previously identified [35]. DMR annotated to *APOB* associated with CN to MCI conversion status (called by both comb-p and DMRcate) was also implicated in neuronal survival and maintenance [45] and previously identified as a DMR in hippocampus in AD patients [24].

The most significant DMR was identified in the AD-associated gene *PM20D1* [46, 47], which was associated with both the slope of CDR-SB (called by comb-p) and the MCI to AD conversion status (called by both comb-p and DMRcate). While variants associated with AD have been characterized as both meQTL and eQTL for *PM20D1*, the differential methylation of *PM20D1* promoters in human samples of AD and differential expression of *PM20D1* in mouse models and human samples of AD were reported [47]. *PM20D1* expression is upregulated by AD-related stressors, amyloid-β and reactive oxygen species, and overexpression of *PM20D1* was neuroprotective in cell and primary cultures [46]. *DUSP22* promoter hypermethylation and mRNA downregulation in the hippocampus of AD patients were reported previously [48]. Further, functional experiments demonstrated that shRNA silencing of *DUSP22* caused the loss of tau phosphorylation at Thr231, while overexpression of *DUSP22* increased Thr231 tau phosphorylation via a mechanism mediated by PKA and p38 [48]. Phosphorylation at Thr231 is one of the first phosphorylation events of tau protein in the AD disease process [49]. The hypomethylation of probes in the DMRs annotated to *DUSP22* was associated with CN to MCI conversion status. *DUSP22* also affects CREB activity via PKA; CREB activity, which has been shown to be important for neuronal survival, axonal growth, and synaptic function [50, 51], is altered in AD. A DMR annotated to *CREB5* and *NTRK2* was also associated with the slope of mPACC_trailsB_ (Additional file 2: Table 4A). These genes and *PTH2R*, *SALL3*, and *HOXA4* (DMRs annotated to these genes were also identified in the slope of mPACC_trailsB_ analysis) all harbor CCAAT/enhancer-binding protein (C/EBP) gamma binding motif, and genes with one or more occurrence of the transcription factor binding site V$CEBPGAMMA_Q6 [52] are enriched (Additional file 2: Table 6A). *PTH2R* was identified as a hub gene in a weighted co-expression network analysis using transcriptomic data from the frontal lobe and temporal cortex in patients with vascular dementia or AD [53]. A decrease in dendritic spine numbers and an imbalance between long-term potentiation and depression caused by oligomeric Aβ, can lead to impaired synaptic transmission [51]. Furthermore, through its binding to p75 neurotrophin receptor (p75^NTR^) and brain-derived neurotrophic factor receptor (encoded by *NTRK2*), Aβ can further worsen the situation [51]. Levels of N-cadherin, a synaptic adhesion molecule, are elevated in the plasma, brain, and CSF of patients with AD [54]. Interestingly, the DMRs annotated to N-cadherin–like gene cluster were associated with rate of cognitive decline (Additional file 2: Tables 4A and B). Taken together, the DMRs uncovered in this study appear to implicate genes involved in synaptic plasticity, Aβ/tau pathology, and pathways mediated by *DUSP22* and PKA/MAPK kinase that affect CREB and C/EBPγ activities. Even though the same DMRs were identified in brain and blood, the change of methylation pattern appears complex, and the directionality of methylation change was often contradictory between brain and blood.

Interestingly, the DMR annotated to *ACY3* was associated with both the slope of mPACC_trailsB_ and MCI to AD conversion status (called by both comb-p and DMRcate); it was also associated with the slope of mPACC_digit_ and CN to MCI conversion status (called by comb-p alone), together representing a mechanism relevant to disease progression in both stages (CN and MCI). *ACY3* was identified as a gene associated with a microglial gene expression profile [55], which was differentially expressed in P301L-tau transgene model [56]. The single cell RNA-Seq profile confirmed the microglia expression specificity (Additional file 1: Fig. 9A). The role of microglia in AD was recently underscored in a recent meta-analysis of genome-wide association studies [57, 58]. *DUSP22* (Additional file 1: Fig. 9B) and *RPL37* (Additional file 2: Tables 4A, 4B; Additional file 1: Fig. 9C) associated with rate of cognitive decline as measured by the slope of mPACC, and *AMPD3* (Additional file 2: Tables 4C, 4E; Additional file 1: Fig. 9D) associated with the slope of CDR-SB and MCI to AD conversion status are also abundantly expressed in microglia. Astrocytes play important roles in CNS, including defense against oxidative stress, mitochondria biogenesis, tissue repair and neurogenesis [59]. *GABBR1 (*Additional file 1: Fig. 9E), *ATP6V0E2* (Additional file 2: Tables 1, 4A; Additional file 1: Fig. 9F), *NWD1* (Additional file 2: Table 4D; Additional file 1: Fig. 9G), and *FGFR2* (Additional file 2: Table 4E; Additional file 1: Fig. 9H) are abundantly expressed in astrocytes. Both *GABBR1* and *FGFR2* are implicated in neurogenesis, learning, and memory [60, 61].

Multiple other DMRs, including *FGFR2* for conversion status in patients with MCI, were detected. The FGF7/FGFR2/PI3K/Akt pathway has been implicated in microRNA-107-induced increased cell proliferation and reduced cell inflammation and apoptosis in an in vitro model of AD [62]. A DMR annotated to *LMTK3* was associated with the rate of cognitive decline as measured by the slope of CDR-SB in patients with MCI, and hypomethylation was associated with faster rate of cognitive decline. Probes annotated to *LMTK3* have been reported to be differentially methylated in the cortex in patients with Down syndrome [63] and in both the dorsal motor nucleus of the vagus and the substantia nigra in patients with Parkinson disease [64]. In Tg4-42 mouse, which expresses N-truncated Aβ_4–42_ and exhibits neuronal loss and behavioral deficits but no plaque formation, the *LMTK3* transcript was reported to be differentially downregulated in aged animals [65]. A related family member, *LMTK2,* which is considered to play a role in neurodegeneration possibly via mechanisms such as tau hyperphosphorylation, enhanced apoptosis, and disrupted axonal transport, was similarly downregulated in an AD animal model [66].

This study has some limitations. Although we replicated the *GABBR1* DMR association with cognitive change from the AddNeuroMed study [33], the overall effect size correlation was weak. This may be related to a relatively small sample size in the compared study (38 MCI to AD converters and 67 nonconverters). In addition, the phenotype of conversion in this study was not limited to a one-year follow-up period, although the converters and nonconverters had generally similar follow-up periods, with a slightly longer follow-up for nonconverters. Comparison with the MADT study was limited to 12 reported DMPs with *P* < 1 × 10^−5^. In addition, the study designs were different (monozygotic twin vs. population study), and the samples were different (the follow-up sample in the MADT study associated with the cognitive change in the preceding 10 years vs. baseline sample in this study associated with the subsequent cognitive change in approximately 5 years). In the present study, the rate of cognitive decline was computed using a linear model (robust regression), which is different from the nonlinear trajectory of cognitive decline previously proposed in a hypothetical model [67]. Even though the motivation for this study was to identify biomarkers for disease progression, we did not find any individual CpG that crossed study-wide significance threshold and had effect size large enough to potentially serve as a prognostic biomarker. A panel of CpGs predictive of disease progression at the individual level cannot be ruled out completely; however, building a generalizable predictive model based on a biosignature would require identification of a set of reliable CpGs associated with disease progression. The sample size in this study for each group was modest after applying the patient population selection criteria, which limits the statistical power for the analysis. The study was conducted on bulk tissue using whole blood or buffy coat. Cell type-specific methylation difference could be obscured in this assay. Replication is needed to confirm the results presented here.

In conclusion, this study replicated multiple DMPs and DMRs and identified additional candidate DMPs and DMRs in peripheral blood that were associated with the rate of cognitive decline in participants in the longitudinal ADNI cohort. The DMRs uncovered in this study implicated genes involved in synaptic plasticity and Aβ/tau pathology and pathways mediated by *DUSP22* and PKA/MAPK kinase and impacting CREB and C/EBPγ activities. Future meta-analysis with additional study datasets will be helpful in prioritizing the reliable findings that can be generalized across studies.

## Methods

### Alzheimer’s disease neuroimaging initiative

Data analyzed in this study were obtained from the ADNI database [68]. The ADNI was launched in 2003 by the National Institute on Aging, the National Institute of Biomedical Imaging and Bioengineering, the United States Food and Drug Administration, private pharmaceutical companies, and nonprofit organizations as a $60 million, 5-year public–private partnership. The primary goal of the ADNI study has been to test whether serial magnetic resonance imaging, positron emission tomography, other biological markers, and clinical and neuropsychological assessments can be combined to measure the progression of MCI and early AD. The data from ADNIMERGE R package, dated September 14, 2018, together with epigenetic data and REGISTRY.csv file (http://adni.loni.usc.edu) were used for the analyses reported here. The ADNIMERGE table merges several key variables from various case report forms and biomarker lab summaries across the ADNI protocols (ADNI1, ADNIGO, and ADNI2).

### DNA methylation profile

DNA methylation profiles were generated at AbbVie, Inc. from blood samples of ADNI participants using Infinium® MethylationEPIC BeadChip (Illumina, Inc., San Diego, CA, USA). Genomic DNA samples obtained from National Cell Repository for Alzheimer’s Disease were bisulfite-converted using the EZ-DNA Methylation Kits (Zymo Research, Irvine, CA, USA) and subsequently analyzed using the Illumina Infinium® HD methylation protocol on the HiScan™ system (Illumina, Inc). Detailed sample-level quality control was described previously [20]. A probe-level quality control was performed as well; probes that did not perform well (probes with detection *P* value ≥ 0.05 in ≥ 1% samples [*n* = 3047] and probes with bead count < 3 in ≥ 5% of samples [*n* = 1011]) were filtered out. A total of 863,718 probes were used in the downstream analysis. Methylation profiles from 653 baseline DNA samples were used in this study. Participants with reverse conversion (e.g., from CN to MCI and back to CN) or conversion from CN to AD, those with a diagnosis of AD at baseline, and those without post-baseline measurements were excluded, leaving a total of 519 samples reported in the study.

### Disease progression phenotype definition

The DNA samples were not necessarily collected at the same time point as the baseline visit for the main ADNI clinical study. Considering that the ADNI epigenetic dataset is a longitudinal analysis of DNA samples, up to 5 samples per participant were available, and the sample from the earliest time point was considered the baseline data. Participants were required to have a follow-up clinical assessment from at least one post-baseline visit.

Disease progression from the baseline of this study was defined using two continuous endpoints for CN participants and one continuous endpoint for patients with MCI; both groups also had a dichotomized endpoint (presence or absence of conversion). The original PACC, which was designed to capture 3 key domains of episodic memory, executive function, and orientation, is a composite of 4 component scores: total recall score, delayed recall score, Digit Symbol Substitution Test (DSST) score, and MMSE total score [26]. The ADNIMERGE database includes a modified PACC (mPACC_trailsB_; a standardized *Z*-score composite of MMSE, logical memory delayed recall, Alzheimer Disease Assessment Scale-Cognitive Subscale delayed word recall, and Trail-Making Test B [27, 69]) and an additional variant of the modified PACC that uses the original component score for DSST (mPACC_digit_) [70]; both mPACC_trailsB_ and mPACC_digit_ were used in this study. For the continuous endpoints, the rate of cognitive decline was measured by the slopes of mPACC_digit_ [26, 71] and mPACC_trailsB_ [72] in CN participants and by the slope of CDR-SB in patients with MCI [73]. For the dichotomized endpoint, a converter status was assigned if there was a conversion of clinical diagnosis (from CN to MCI or from MCI to AD) at a post-baseline visit. If the diagnosis was undefined at the DNA sampling visit, but the clinical diagnosis had been consistent before and after the DNA sampling visit, the diagnosis at DNA sampling was assumed to be the same as that at the adjacent visits.

### Identification of DMPs

*M-value*, which provides higher detection rates and true positive rates for both highly methylated and unmethylated CpG sites and is considered statistically more valid than *beta-value* [74], was used to identify DMPs using R package *limma* [75]. The statistical model, adjusted for age at DNA sampling, sex, cell composition, DNA source (buffy coat vs. whole blood), was used to analyze rate of cognitive decline or conversion status. Since the study was prone to significant inflation and bias of test statistics, we applied a Bayesian method in R package BACON v1.10.1 to control for inflation of test statistics and for lambda inflation factors before and after correction were reported; the Bayesian method is based on estimation of the empirical null distribution [31]. A stringent threshold using Bonferroni correction was used to declare study-wide significance. The discovered DMPs for each endpoint were assessed for consistency in 2 ways: (1) the effect size and directionality were compared between the continuous and dichotomized endpoints, and (2) the effect sizes from this study were compared with those reported in a recent meta-analysis in brain [35] and several EWAS studies in blood [20, 34], recognizing that the phenotype in the two studies was different.

### Identification and annotation of DMRs and genomic feature enrichment

DMRs were identified using comb-p [76] with a distance of 500 bp and a seeded *P* value of 1.0 × 10^−4^. The DMR analyses were carried out for all probes, irrespective of the directionality of the differential methylation, and DMRs with at least 3 probes and Sidak corrected *P* < 0.05 were considered significant and are reported. Comb-p identifies regions enriched for probes with low *P* values using the Stouffer-Liptak method to correct for autocorrelation and adjusts for multiple testing using the Sidak correction. The identified DMRs were annotated by HOMER software [77]. HOMER first determines the distance of a DMR to the nearest transcription start site and assigns the DMR to that gene, and it determines the genomic annotation of the region occupied by the center of the DMR.

To provide additional evidence for the DMR analysis, a secondary DMR calling algorithm DMRcate was employed [78]. The algorithm used the default parameters, except for the following: the robust regression for *limma* was used to be consistent with the DMP analysis, the test statistics after BACON statistics were used with a pcutoff at 1.0 × 10^−4^, and a minimum of 3 CpG probes were applied for DMR calling. DMRcate calculates two smoothed estimates (one based on the square of moderated *limma* t statistic and the other null model) using a Satterthwaite approximation to compare these estimates and adjusts for multiple testing using the FDR method. Both sets of DMR results are reported and compared for consistency, but the comb-p analysis was considered as the primary analysis.

The identified DMRs were also compared to 2 lists of DMRs reported previously to be associated with conversion to AD [25, 33].

### Gene set enrichment and overrepresentation analyses

Using R package, methylGSA, GSEA analysis was conducted that used Robust Rank Aggregation to adjust for multiple *P* values of each gene and applied pre-ranked version of GSEA in gene set testing [79, 80]. The following gene ontology databases were used: subsets of Molecular Signatures Database, KEGG database, and c2.cp v7.0 (includes a superset of c2.cp.biocarta, c2.cp.kegg, and c2.cp.reactome among others) [81–83]. Over-representation analysis was performed using R package *missMethyl* [84] to test for pathway enrichment using a hypergeometric test, taking into account the number of CpG sites per gene on the EPIC array.

### Co-occurrence of biological concepts and genes using Euretos AI Platform

Since the database gene assignment to ontology terms could be incomplete, we also applied co-occurrence analysis of genes and key biological concepts, such as neurogenesis, amyloid, tau proteins, and cognition, to identify key genes annotated by DMRs (using the comb-p approach) with prior evidence associated with these terms using Euretos AI Platform (Netherlands). Synonyms were also used in the search; therefore, such analysis could result in false positive findings. With the relationship pairs with 5 or more references, we followed up with a human curation of abstracts, and false positives were flagged.‘

## Supplementary Information

**Additional file 1:** Supplemental Figures.

**Additional file 2:** Supplemental Tables.

## Data Availability

Data used in preparation of this article were obtained from the ADNI database (adni.loni.usc.edu). ADNI data are disseminated by the Laboratory for Neuro Imaging at the University of Southern California.

## Abbreviations

AD: Alzheimer’s disease
ADNI: Alzheimer’s Disease Neuroimaging Initiative
CDR-SB: Clinical Dementia Rating Scale Sum of Boxes
CN: Cognitively normal
CpG: Cytosine-phosphate-guanine
CSF: Cerebrospinal fluid
DMP: Differentially methylated position
DMR: Differentially methylated region
EWAS: Epigenome-wide association study
MCI: Mild cognitive impairment
MMSE: Mini-Mental State Examination
mPACC: Modified Preclinical Alzheimer Cognitive Composite
mPACC_digit_: MPACC that used Digit Symbol Substitution Test
mPACC_trailsB_: MPACC that used Trail-Making Test Part B
mQTL: Methylation quantitative trait locus
NCRAD: National Centralized Repository for Alzheimer's Disease and Related Dementias
SNP: Single nucleotide polymorphism
