# Supplementary material for: Association of peripheral blood DNA methylation level with Alzheimer’s disease progression
Source: Clin Epigenetics. 2021 Oct 15;13:191. doi: 10.1186/s13148-021-01179-2 (PMC8518178; doi:10.1186/s13148-021-01179-2)
Supplement: Supplementary file 1 — Additional file 1: Supplemental Figures. [file 13148_2021_1179_MOESM1_ESM.docx]

**Supplemental Figures**

**Association of Peripheral Blood DNA Methylation Level with Alzheimer’s Disease Progression**

Qingqin S. Li, Aparna Vasanthakumar, Justin W. Davis, Kenneth B. Idler, Kwangsik Nho, Jeffrey F. Waring, Andrew J. Saykin_,_ for the Alzheimer’s Disease Neuroimaging Initiative (ADNI)

**Table of Contents**

[**Supplemental Figure 1.** Q-Q plots for the EWAS before and after BACON correction. 3](#_Toc77669641)

[**(1A)** Results of DMP analysis of slope of mPACC_trailsB_ 3](#_Toc77669642)

[**(1B)** Results of DMP analysis of slope of mPACC_digit_. 3](#_Toc77669643)

[**(1C)** Conversion status for the CN participants (converters vs. nonconverters). 4](#_Toc77669644)

[**(1D)** Slope of CDR-SB. 4](#_Toc77669645)

[**(1E)** Conversion status for patients with MCI (converters vs. nonconverters). 5](#_Toc77669646)

[**Supplemental Figure 2.** Manhattan plots for the EWAS after BACON correction. 6](#_Toc77669647)

[**(2A)** Slope of mPACC_trailsB._ 6](#_Toc77669648)

[**(2B)** Slope of mPACC_digit._ 7](#_Toc77669649)

[**(2C)** Conversion status for the CN participants (converters vs nonconverters). 8](#_Toc77669650)

[**(2D)** Slope of CDR-SB. 9](#_Toc77669651)

[**(2E)** Conversion status for patients with MCI (converters vs nonconverters). 10](#_Toc77669652)

[**Supplemental Figure 3.** Correlation between mPACC_digit_ and CDR-SB or mPACC_trailsB_. 11](#_Toc77669653)

[**(3A)** Correlation between mPACC_digit_ and CDR-SB. 11](#_Toc77669654)

[**(3B)** Correlation between mPACC_digit_ and mPACC_trailsB_. 12](#_Toc77669655)

[**Supplemental Figure 4.** Correlation between mPACC_digit_ and CDR-SB. 13](#_Toc77669656)

[**(4A)** Participants who were CN at baseline. 13](#_Toc77669657)

[**(4B)** Patients with MCI at baseline. 14](#_Toc77669658)

[**(4C)** Participants who were CN or had MCI at baseline 15](#_Toc77669659)

[**Supplemental Figure 5.** Correlation of the effect size (ES) in x-axis for the MCI to AD conversion status from this study vs the ES (diagnostic estimate; y-axis) from the 1000 CpG sites associated with MCI to AD conversion status in the AddNeuroMed study (Supplemental Table 5 in [1]). 16](#_Toc77669660)

[**Supplemental Figure 6.** Correlation of the effect size (ES) for the slope of mPACC_trailsB_ and CDR-SB vs the ES for the 220 CpG sites across cortex in the brain previously identified in an epigenome-wide association meta-analysis study. 17](#_Toc77669661)

[**(6A)** Correlation between ES for the slope of mPACC_trailsB_ and ES for the 220 CpG sites across cortex. 17](#_Toc77669662)

[**(6B)** Correlation between ES for the slope of CDR-SB and ES for the 220 CpG sites across cortex. 18](#_Toc77669663)

[(**6C**) Correlation between ES for AD vs CN [2] and ES for the 220 CpG sites across cortex [3]. 19](#_Toc77669664)

[**Supplemental Figure 7.** Correlation of the effect size (ES) for the slope of mPACCtrailsB and CDR-SB vs the ES for AD vs CN from the same ADNI cohort 20](#_Toc77669665)

[**(7A)** Correlation between ES for the slope of mPACC_trailsB_ and ES for AD vs CN. 20](#_Toc77669666)

[**(7B)** Correlation between ES for the slope of CDR-SB and ES for AD vs CN. 21](#_Toc77669667)

[**Supplemental Figure 8.** DMRs were associated with the rate of cognitive decline. 22](#_Toc77669668)

[**(8A)** A DMR annotated to *HOXA4* was associated with the slope of mPACC_trailsB_. 22](#_Toc77669669)

[**(8B)** A DMR annotated to *HOXA4* was associated with the slope of mPACC_digit_. 23](#_Toc77669670)

[**(8C)** The DMRs annotated to *HOXB6* (left) and *HOXB9* (right) were associated with the slope of CDR-SB. 24](#_Toc77669671)

[**(8D)** Expanded *HOXB* gene cluster including the DMRs annotated to *HOXB6*/*HOXB9* (Figure 7C) were associated with the slope of CDR-SB. Pink filled squares denote DMRs and filled circles denote CpG sites. 25](#_Toc77669672)

[**(8E)** The DMRs annotated to *GABBR1* were associated with the MCI to AD conversion status. 26](#_Toc77669673)

[**(8F)** The DMRs annotated to *DUSP22* were associated with the CN to MCI conversion status. 27](#_Toc77669674)

[**(8G)** The DMRs annotated to *LMTK3* were associated with the slope of CDR-SB. 28](#_Toc77669675)

[**Supplemental Figure 9.** Cell type expression profile of (A) *ACY3* (B) *DUSP22* (C) *RPL37* (D) AMPD3 (E) *GABBR1* (F) *ATP6V0E2* (G) *NWD1* (H) *FGFR2.* 29](#_Toc77669676)

[(**9A**) ACY3. 29](#_Toc77669677)

[(**9B**) DUSP22. 29](#_Toc77669678)

[(**9C**) RPL37. 30](#_Toc77669679)

[(**9D**) AMPD3. 30](#_Toc77669680)

[(**9E**) GABBR1. 31](#_Toc77669681)

[(**9F**) ATP6V0E2. 31](#_Toc77669682)

[(**9G**) NWD1. 32](#_Toc77669683)

[(**9H**) FGFR2. 32](#_Toc77669684)

[**References** 33](#_Toc77669685)

# **Supplemental Figure 1.** Q-Q plots for the EWAS before and after BACON correction.

## **(1A)** Results of DMP analysis of slope of mPACC_trailsB_

**
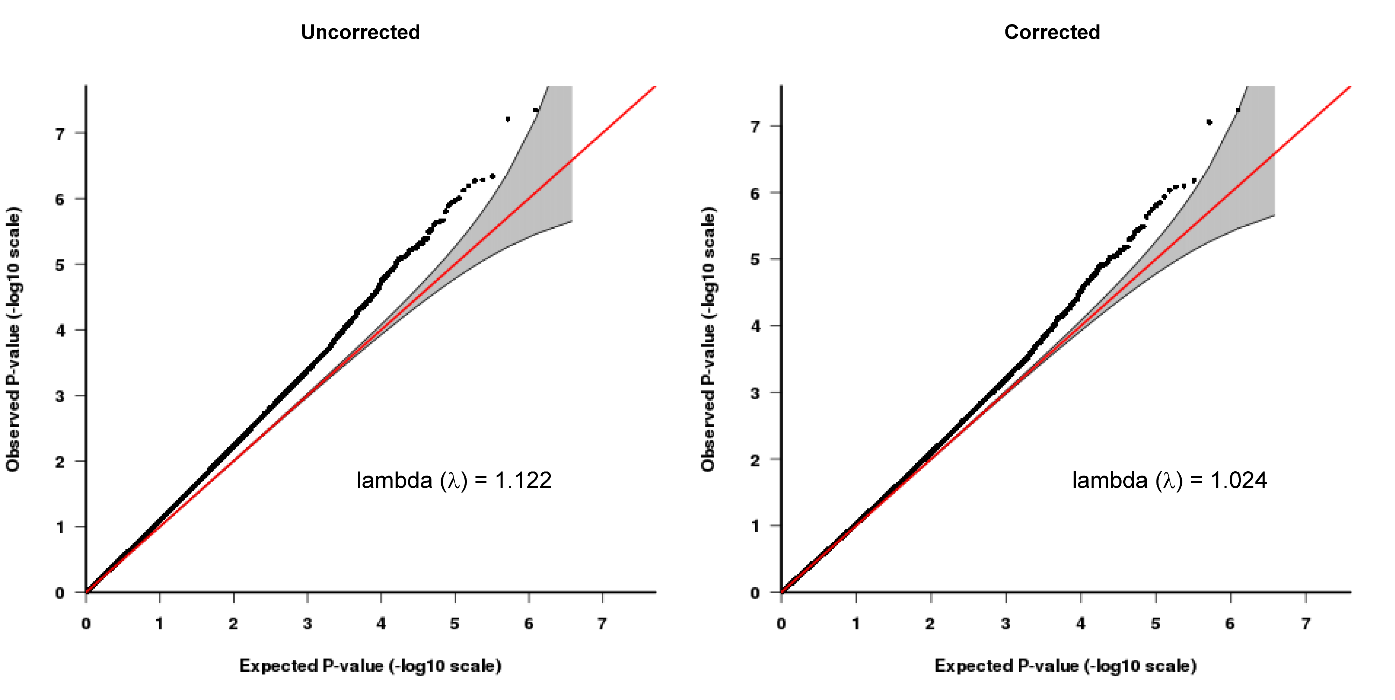
**

## **(1B)** Results of DMP analysis of slope of mPACC_digit_.

**
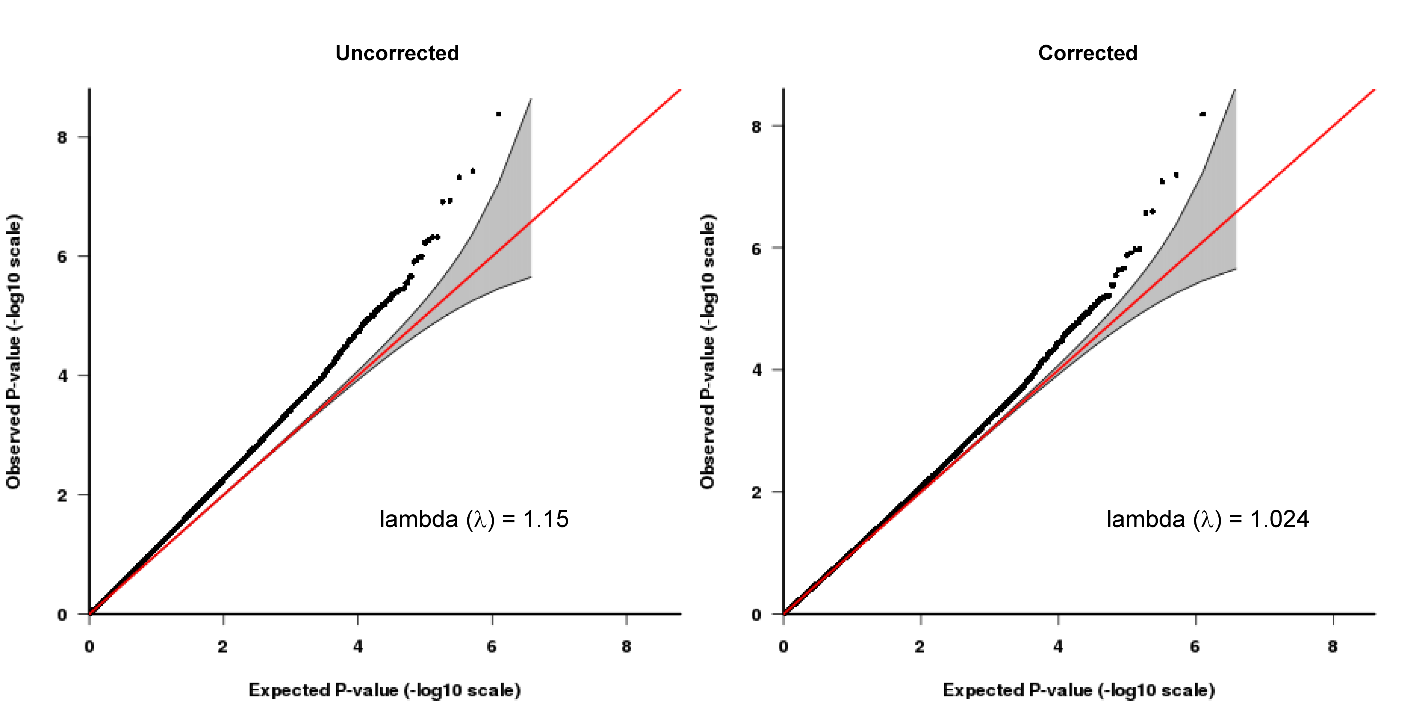
**

## **(1C)** Conversion status for the CN participants (converters vs. nonconverters).

**
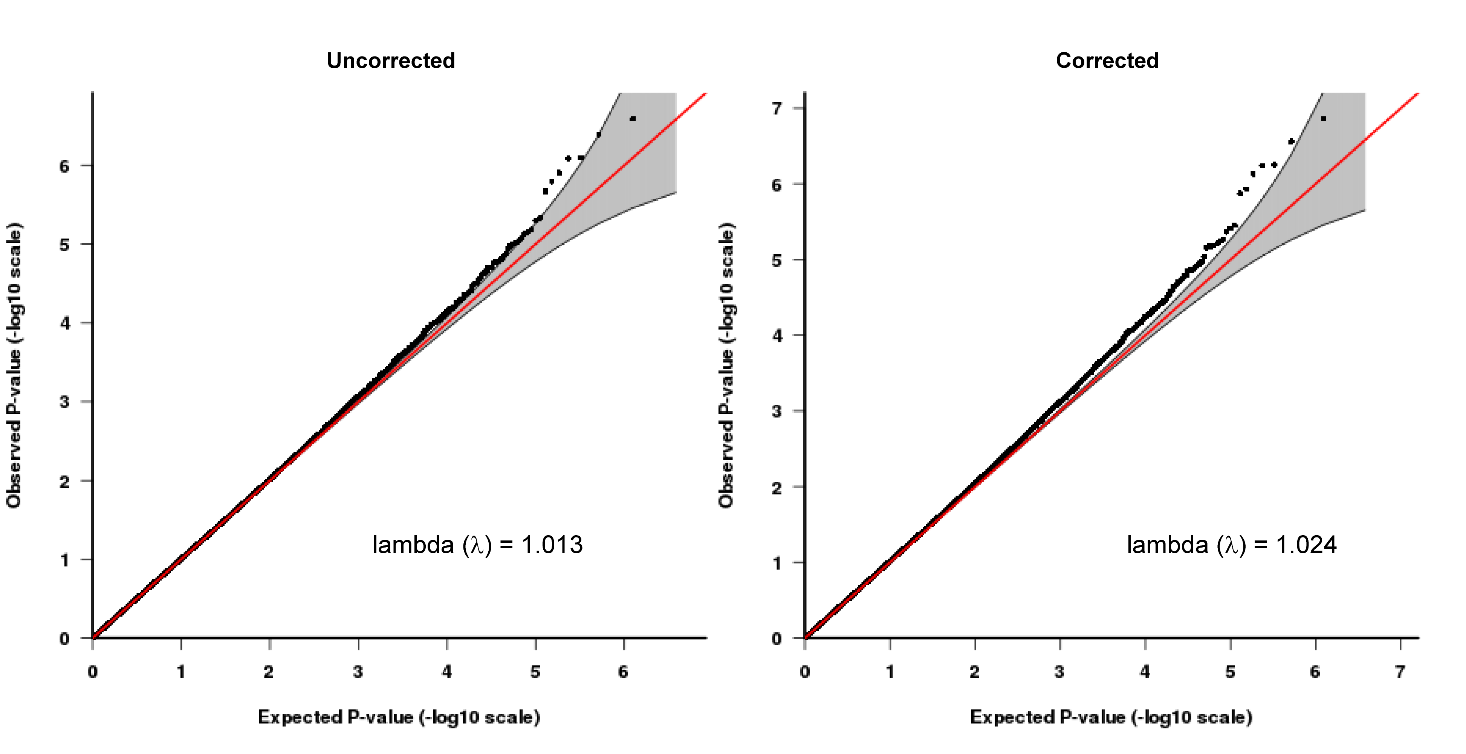
**

## **(1D)** Slope of CDR-SB.

**
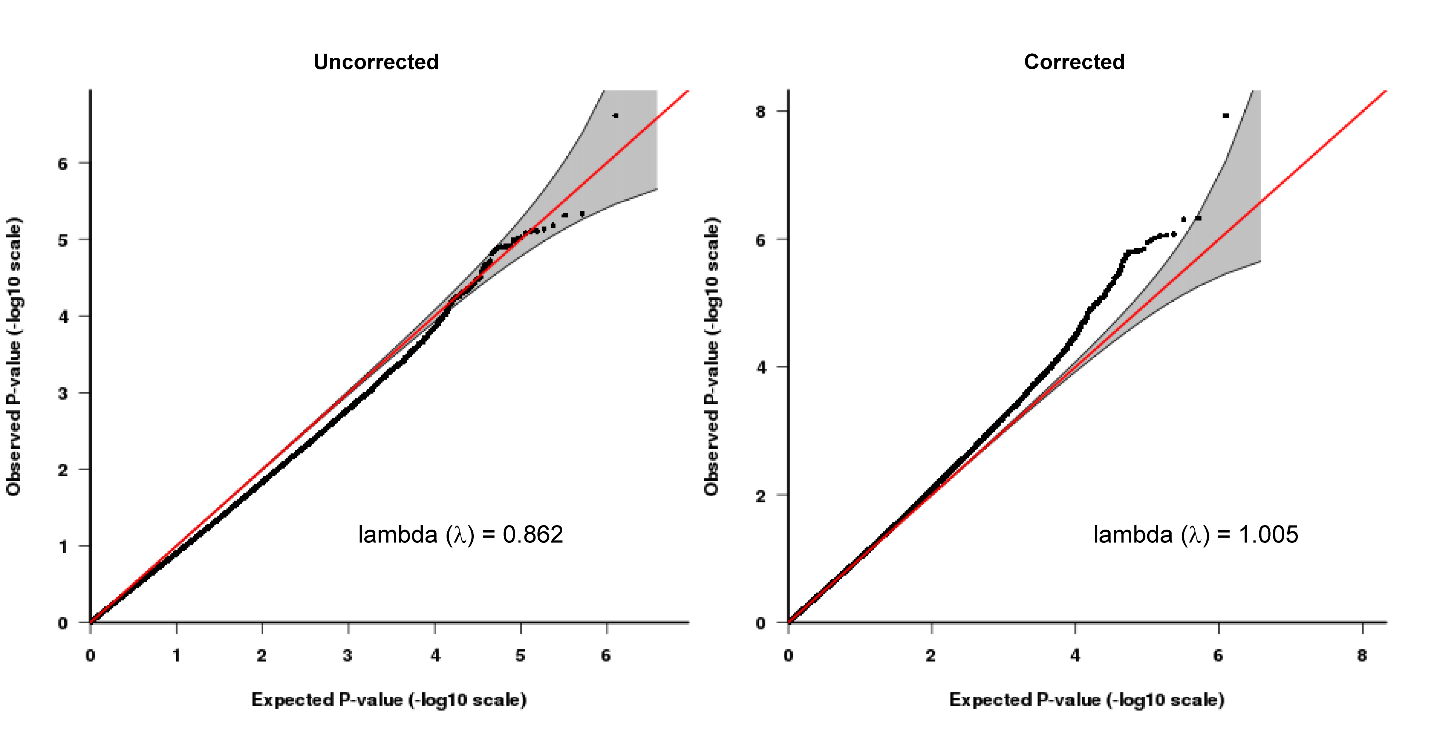
**

## **(1E)** Conversion status for patients with MCI (converters vs. nonconverters).

**
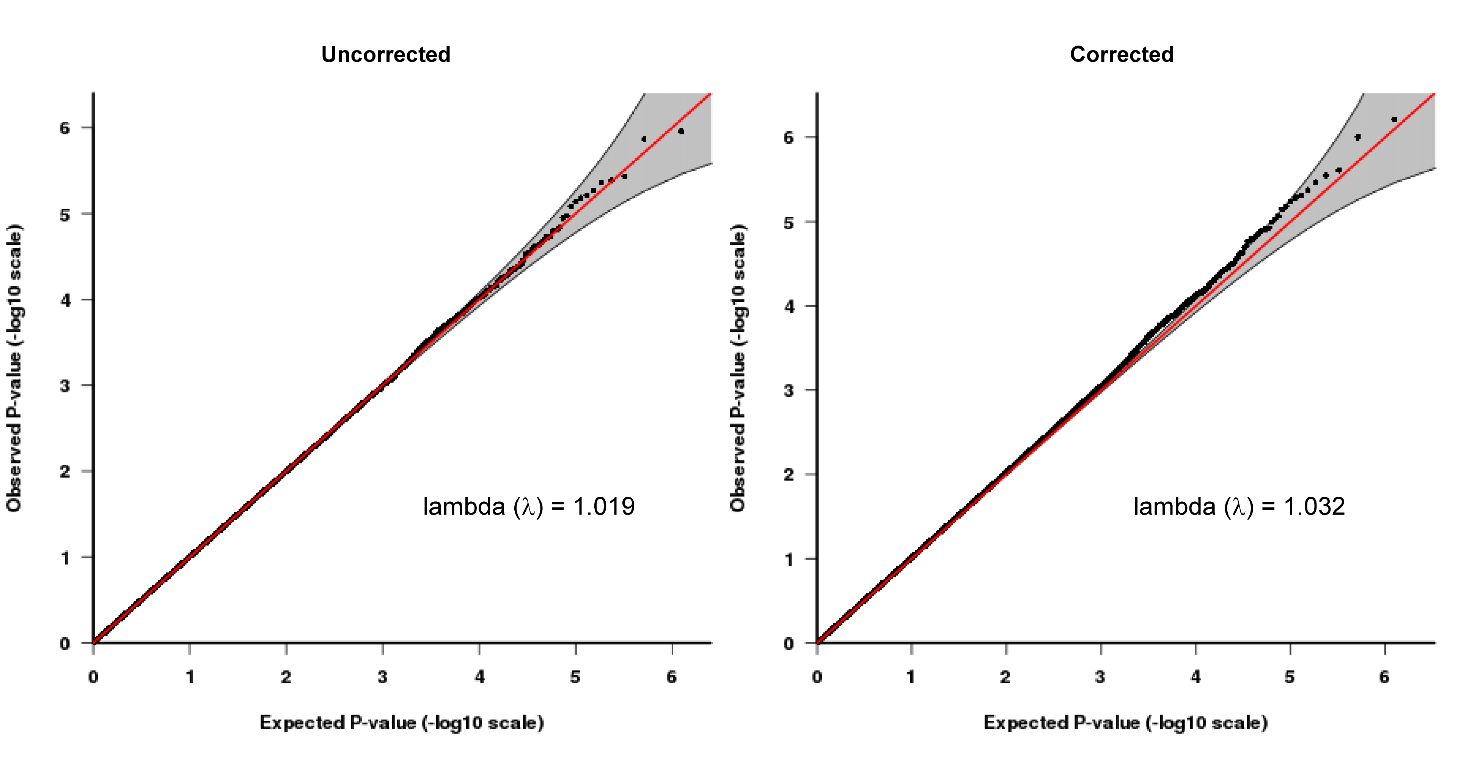
**

# **Supplemental Figure 2.** Manhattan plots for the EWAS after BACON correction.

## _
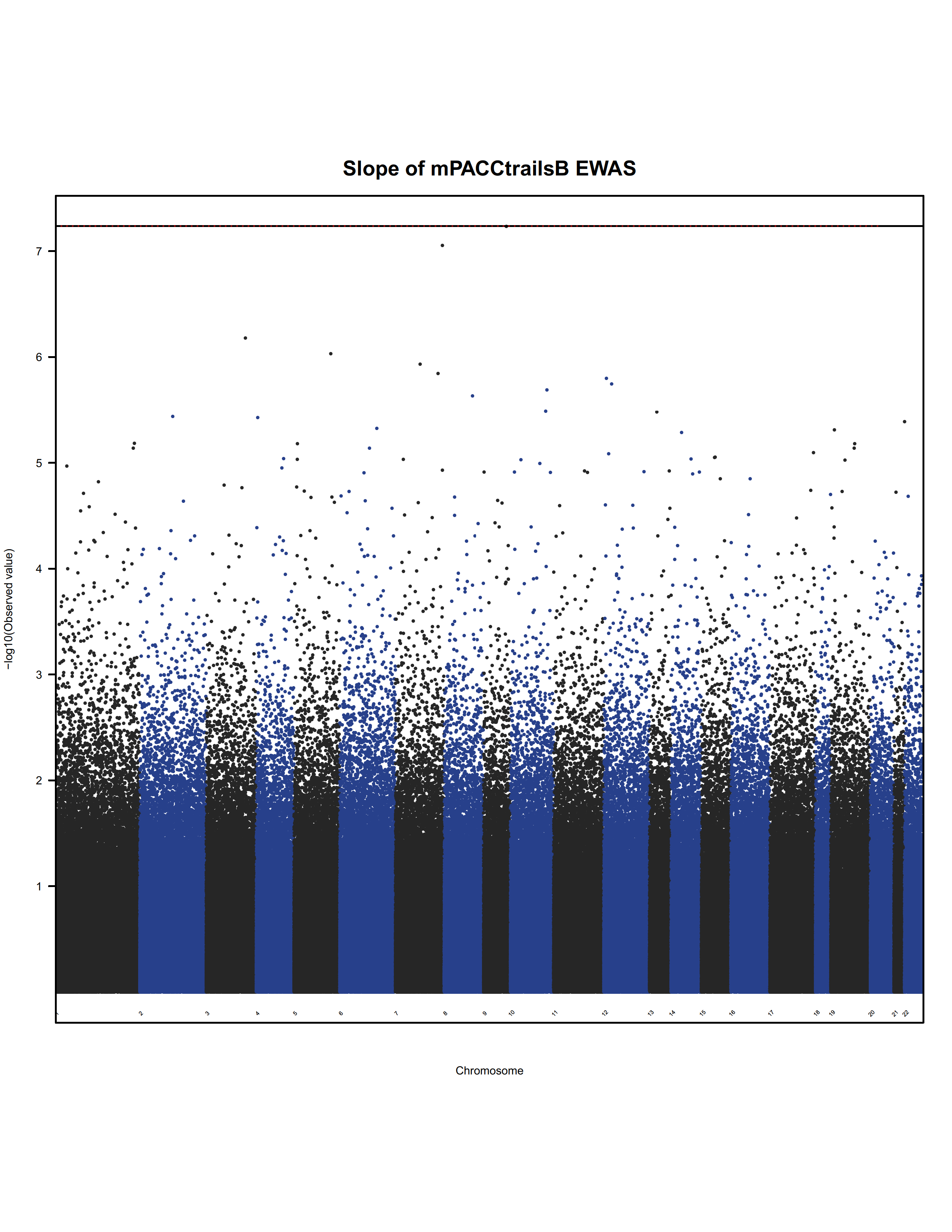
_**(2A)** Slope of mPACC_trailsB._

## **(2B)** Slope of mPACC_digit._

**
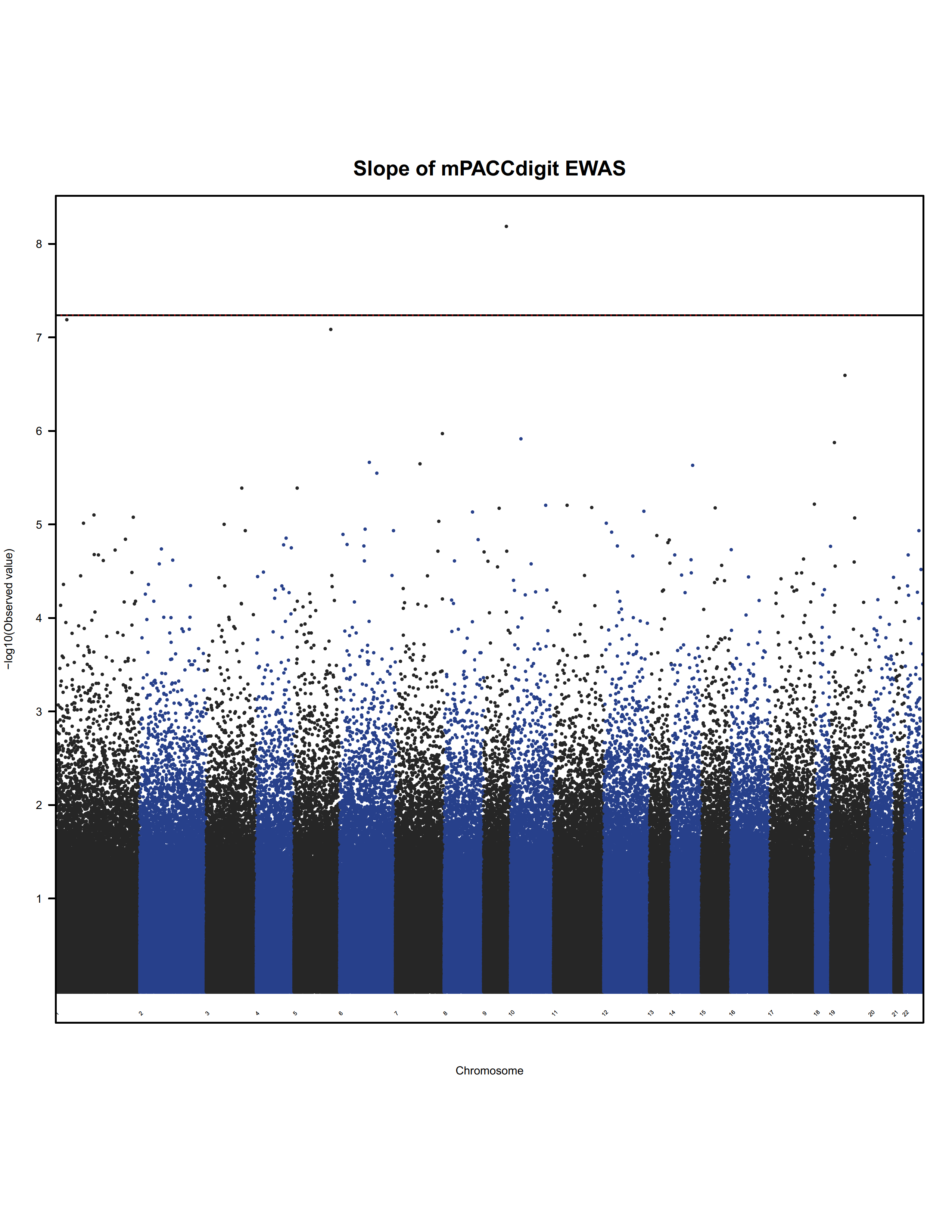
**

## **(2C)** Conversion status for the CN participants (converters vs nonconverters).


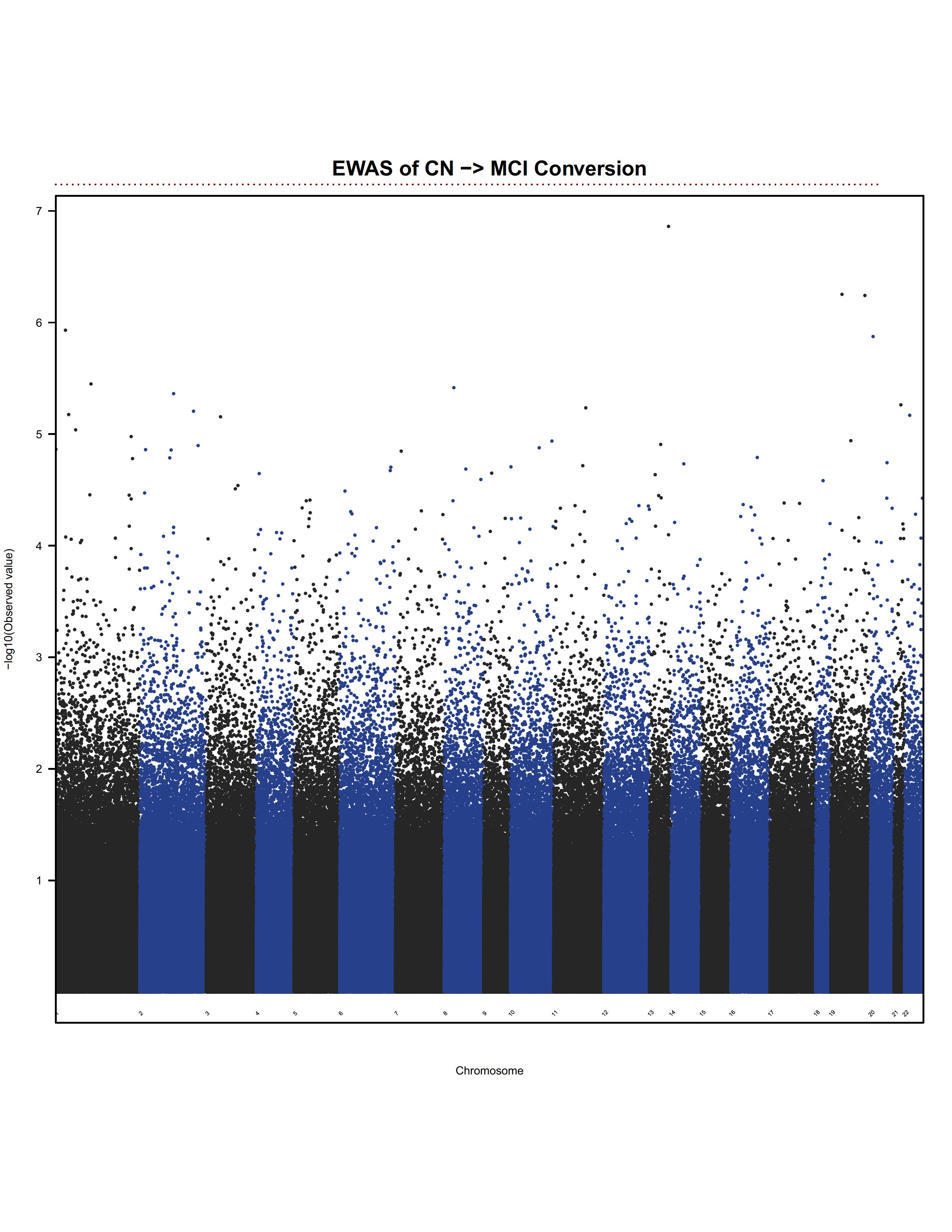


## **(2D)** Slope of CDR-SB.

**
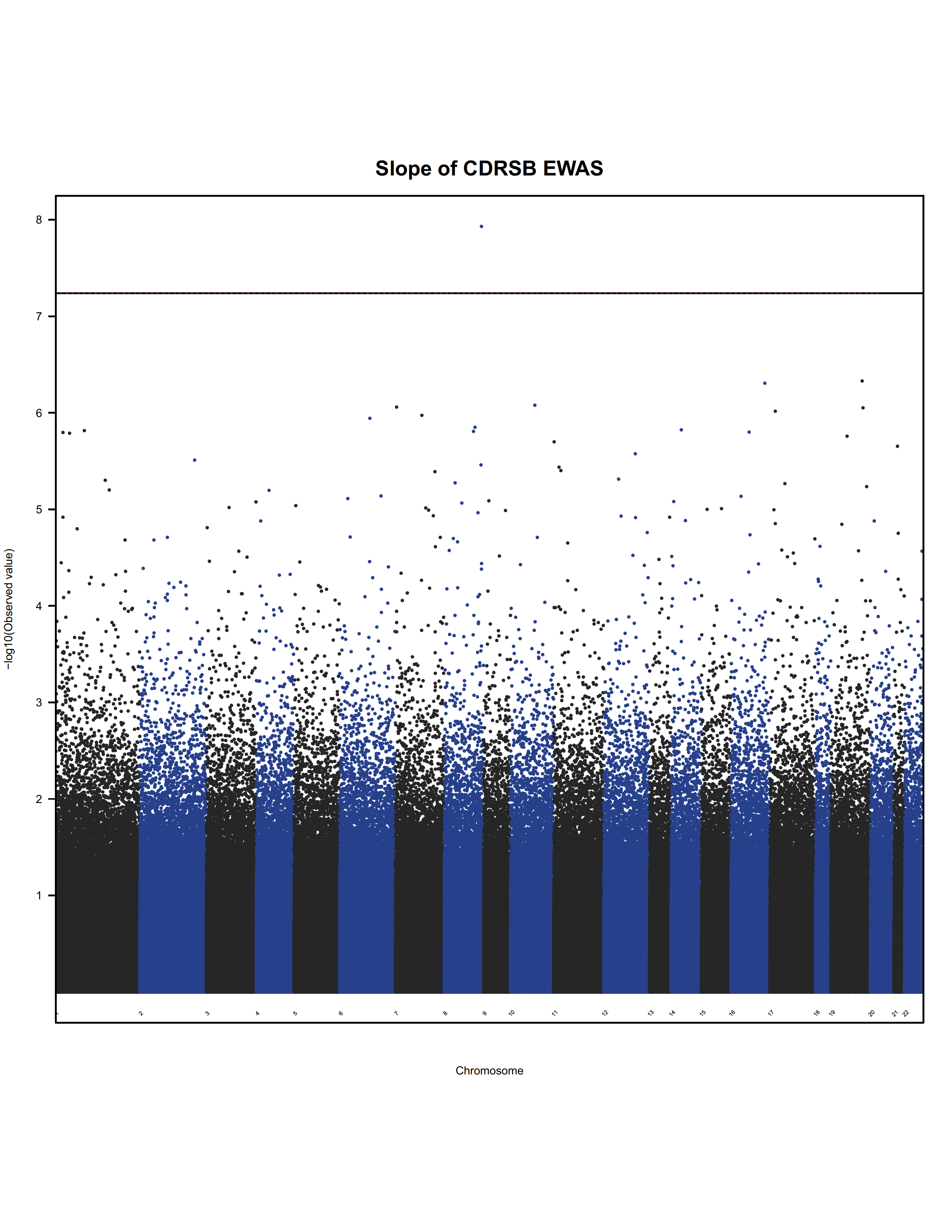
**

## **(2E)** Conversion status for patients with MCI (converters vs nonconverters).

**
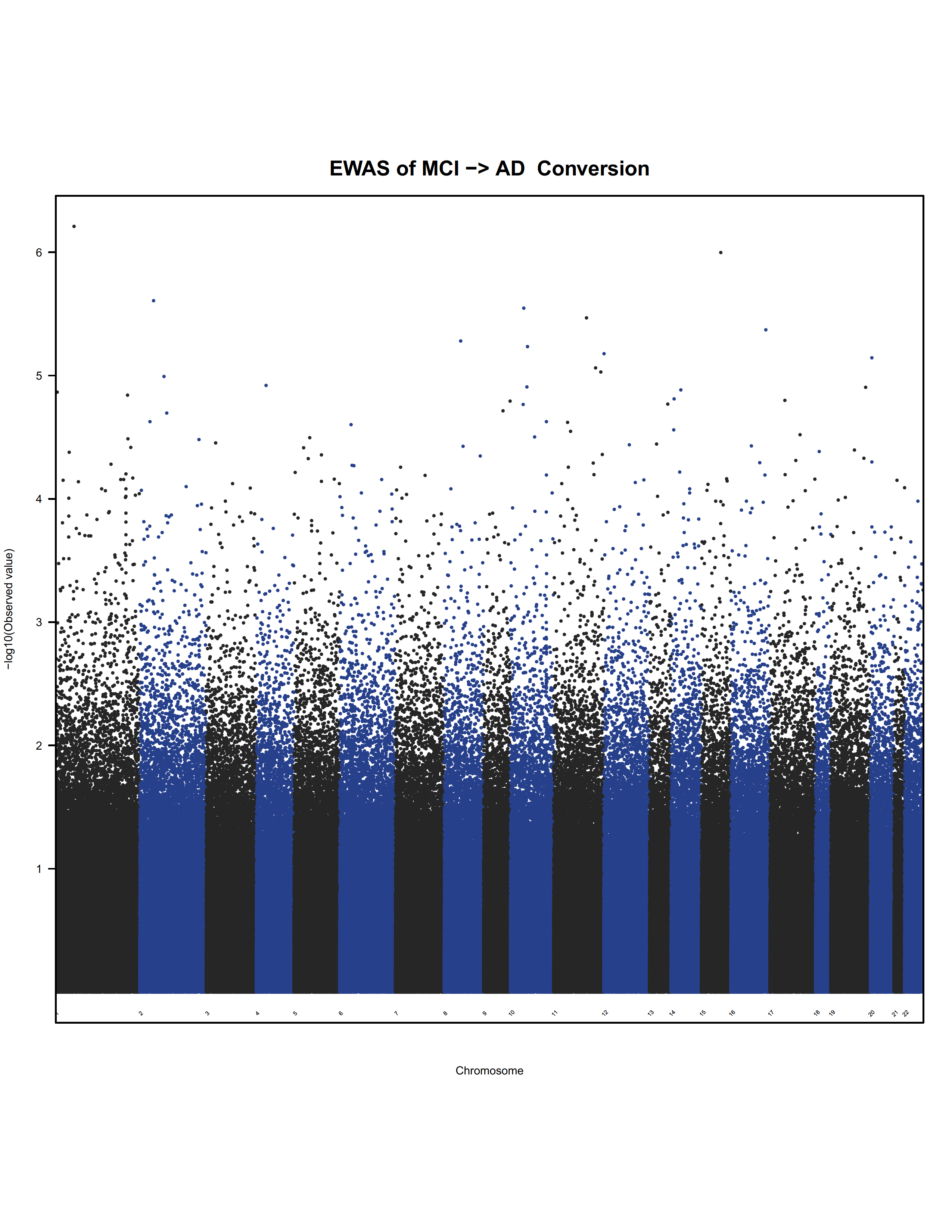
**

# **Supplemental Figure 3.** Correlation between mPACC_digit_ and CDR-SB or mPACC_trailsB_.

## **(3A)** Correlation between mPACC_digit_ and CDR-SB.

**
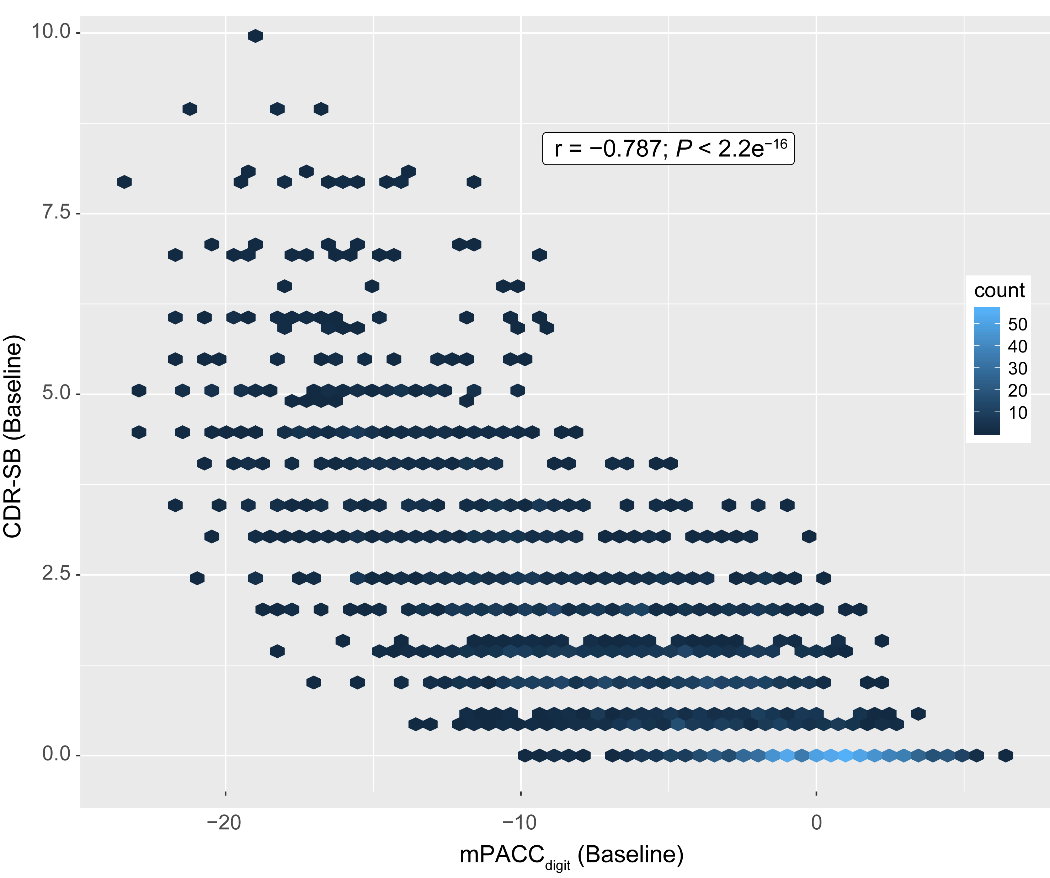
**

## **
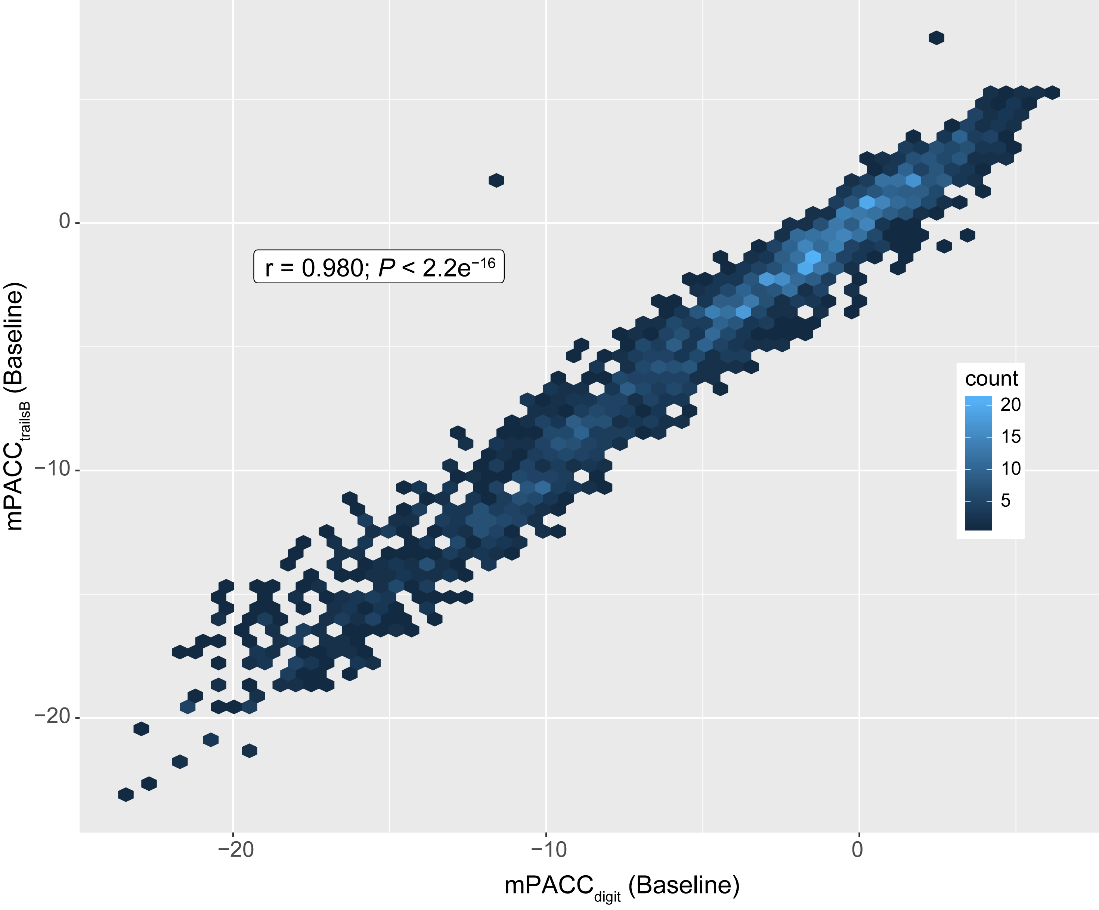
(3B)** Correlation between mPACC_digit_ and mPACC_trailsB_.

# **Supplemental Figure 4.** Correlation between mPACC_digit_ and CDR-SB.

## **(4A)** Participants who were CN at baseline.


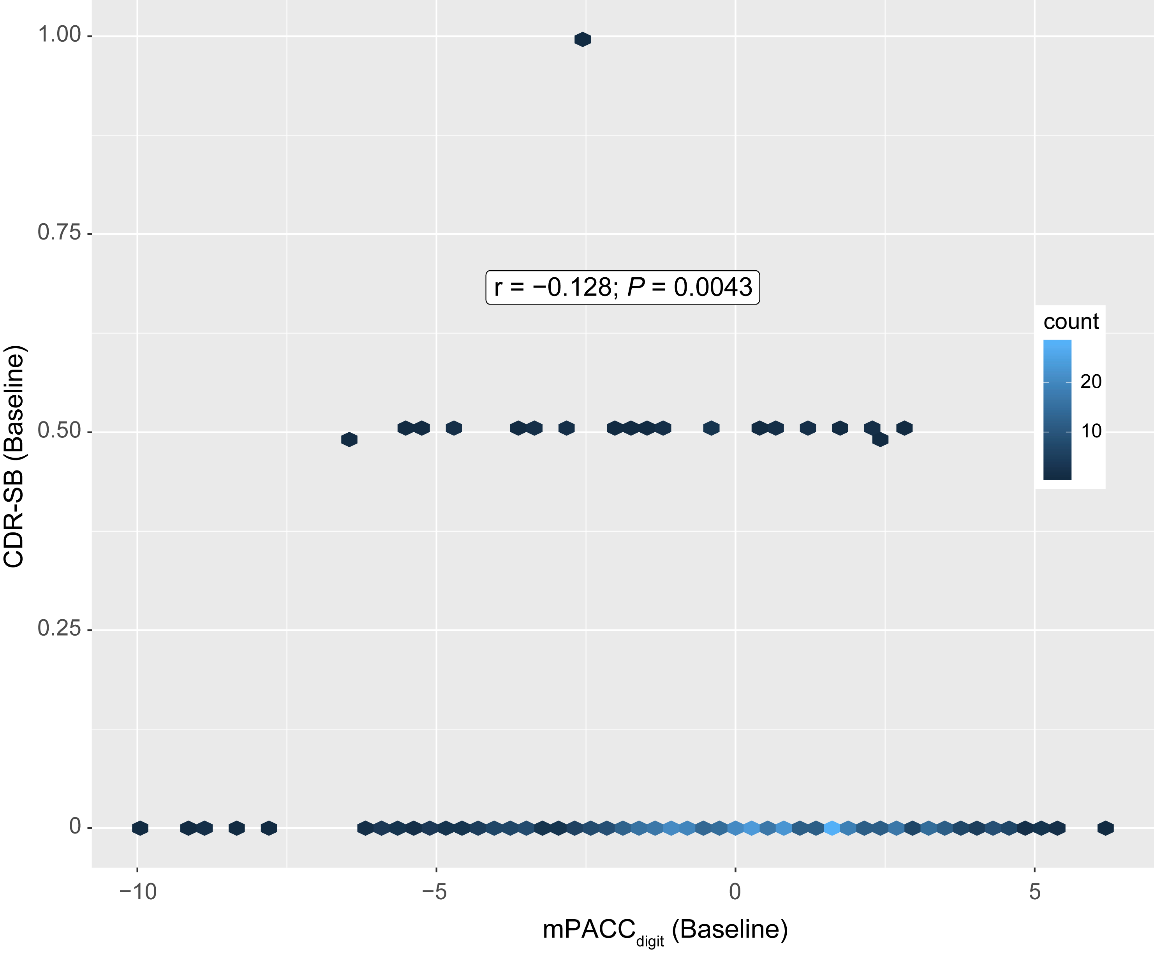


## **(4B)** Patients with MCI at baseline.


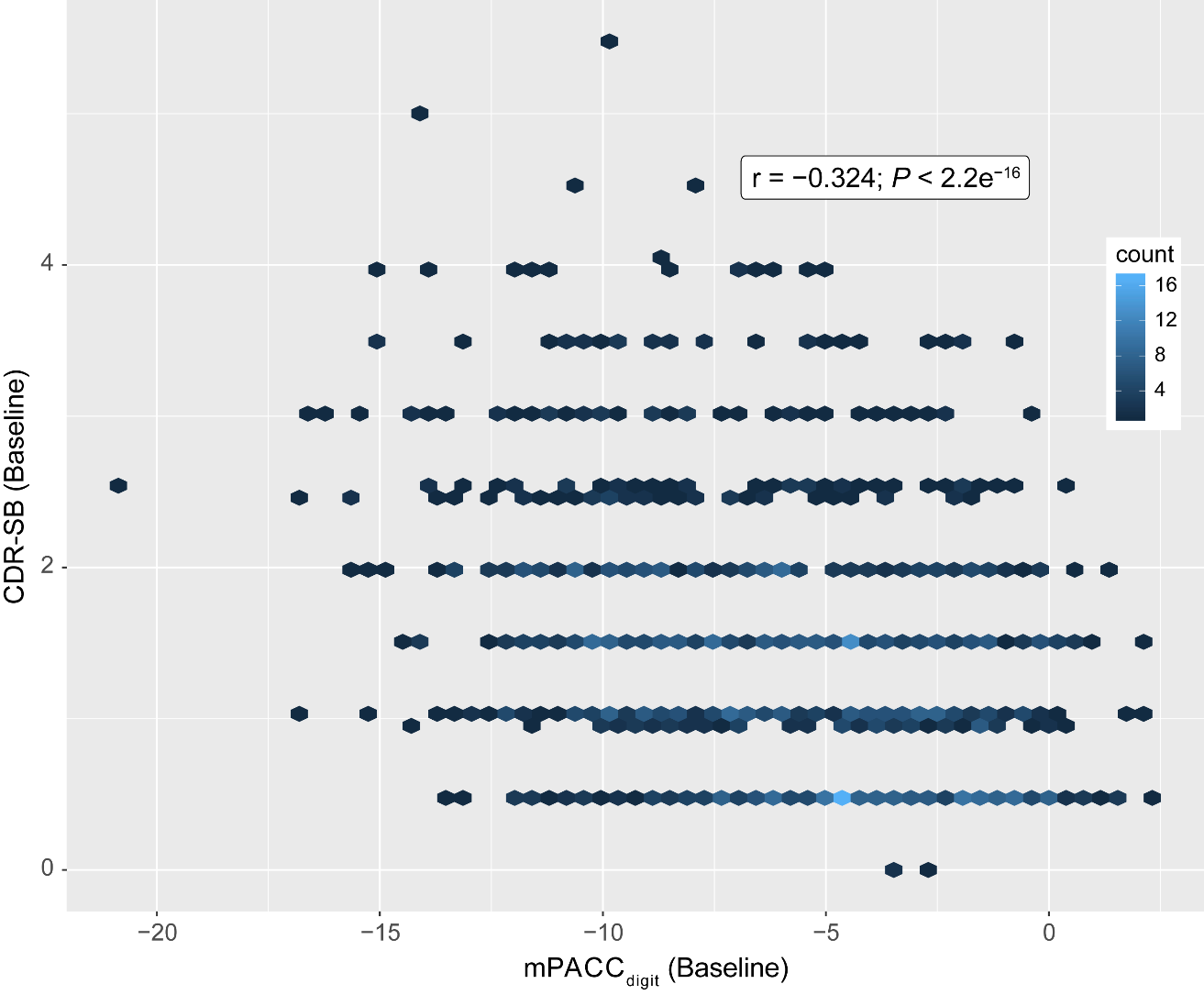


## **(4C)** Participants who were CN or had MCI at baseline


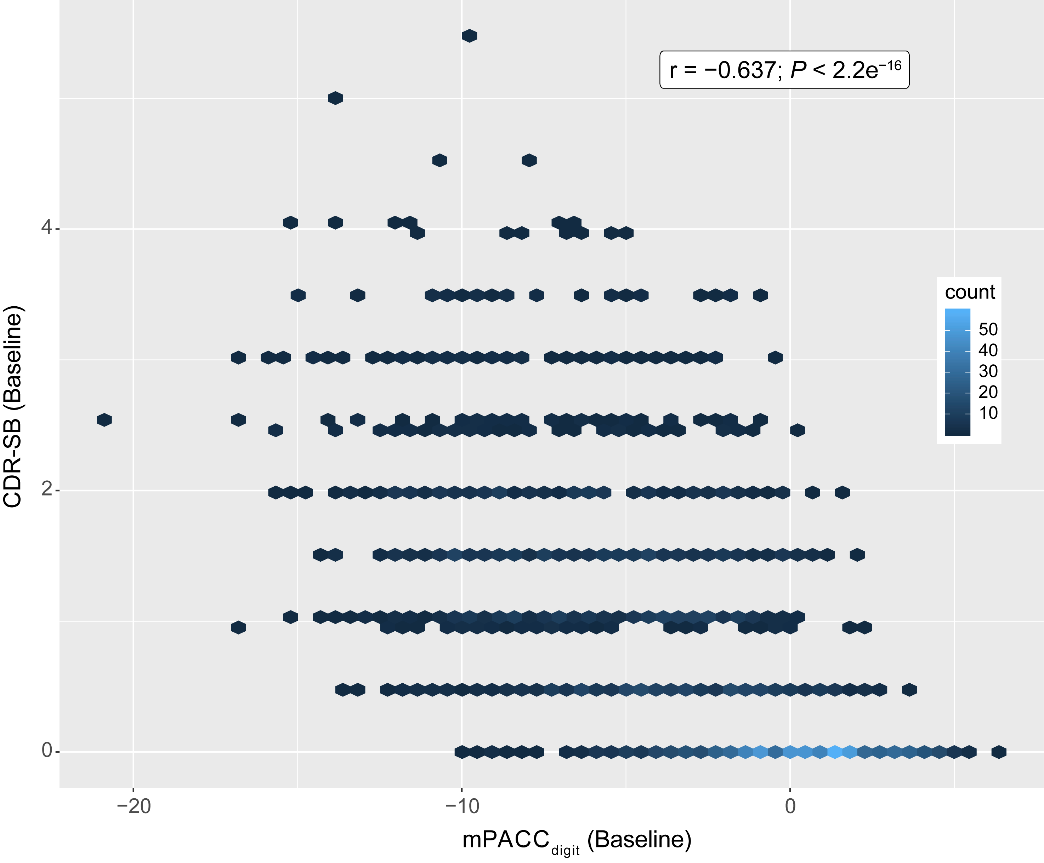


# **Supplemental Figure 5.** Correlation of the effect size (ES) in x-axis for the MCI to AD conversion status from this study vs the ES (diagnostic estimate; y-axis) from the 1000 CpG sites associated with MCI to AD conversion status in the AddNeuroMed study (Supplemental Table 5 in [1]).

**
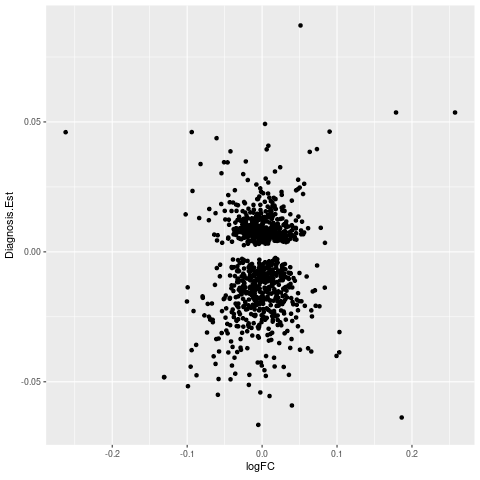
**

# **Supplemental Figure 6.** Correlation of the effect size (ES) for the slope of mPACC_trailsB_ and CDR-SB vs the ES for the 220 CpG sites across cortex in the brain previously identified in an epigenome-wide association meta-analysis study.

## **(6A)** Correlation between ES for the slope of mPACC_trailsB_ and ES for the 220 CpG sites across cortex.





## **(6B)** Correlation between ES for the slope of CDR-SB and ES for the 220 CpG sites across cortex.





## (**6C**) Correlation between ES for AD vs CN [2] and ES for the 220 CpG sites across cortex [3].


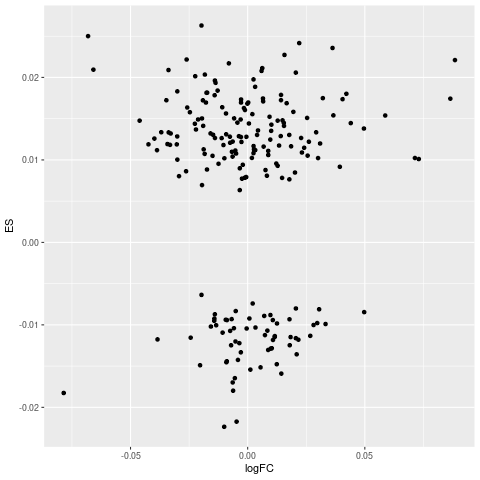


**Supplemental Figure 7.** Correlation of the effect size (ES) for the slope of mPACCtrailsB and CDR-SB vs the ES for AD vs CN from the same ADNI cohort [2]

## **(7A)** Correlation between ES for the slope of mPACC_trailsB_ and ES for AD vs CN.


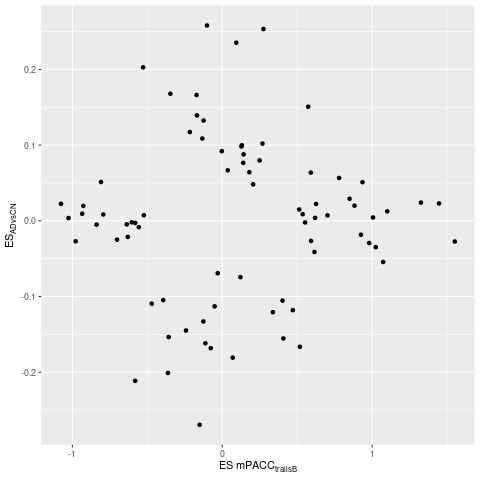


## **(7B)** Correlation between ES for the slope of CDR-SB and ES for AD vs CN.


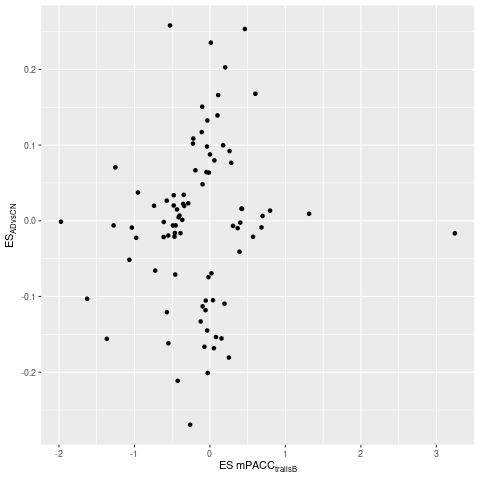


# **Supplemental Figure 8.** DMRs were associated with the rate of cognitive decline.

## ***
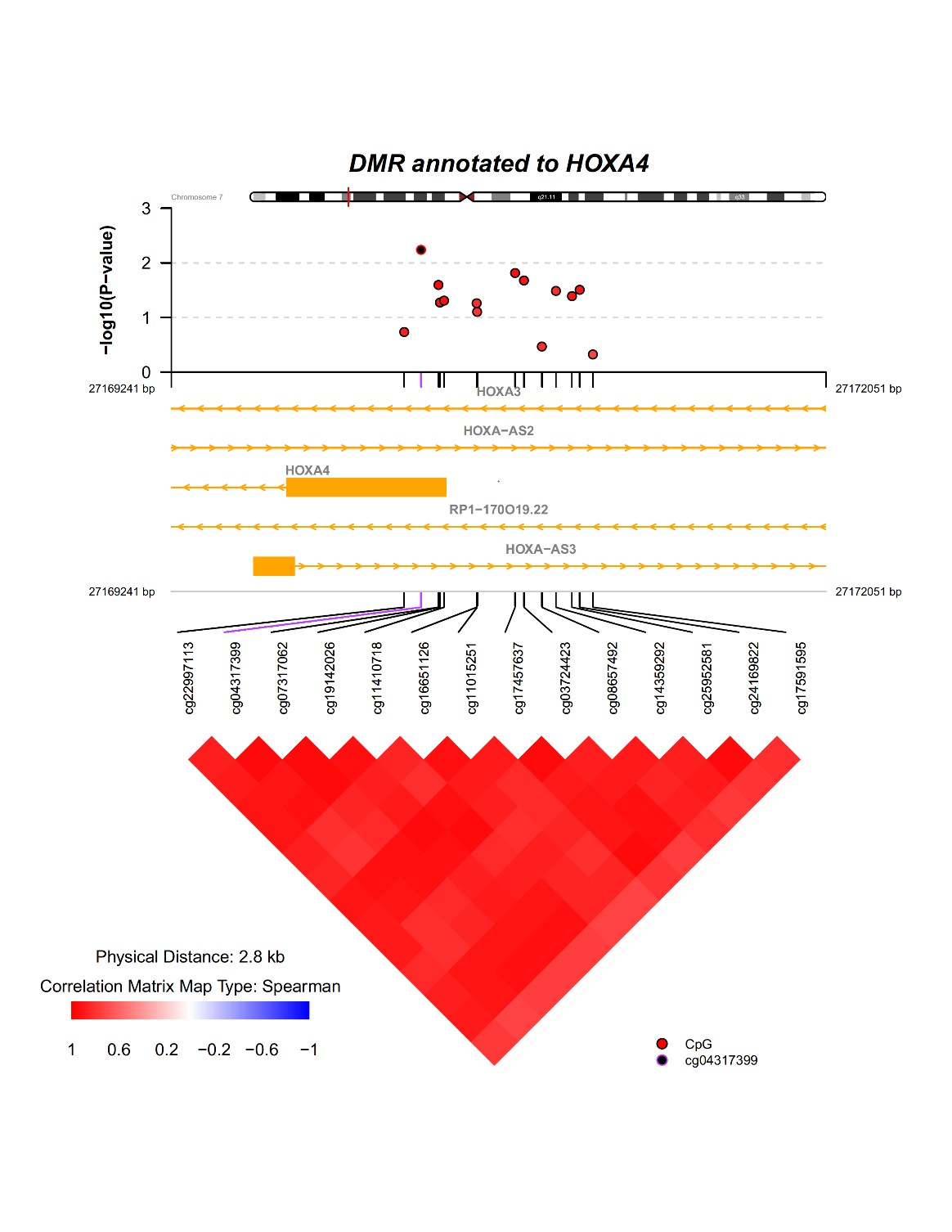
*(8A)** A DMR annotated to *HOXA4* was associated with the slope of mPACC_trailsB_.

## **(8B)** A DMR annotated to *HOXA4* was associated with the slope of mPACC_digit_.

***
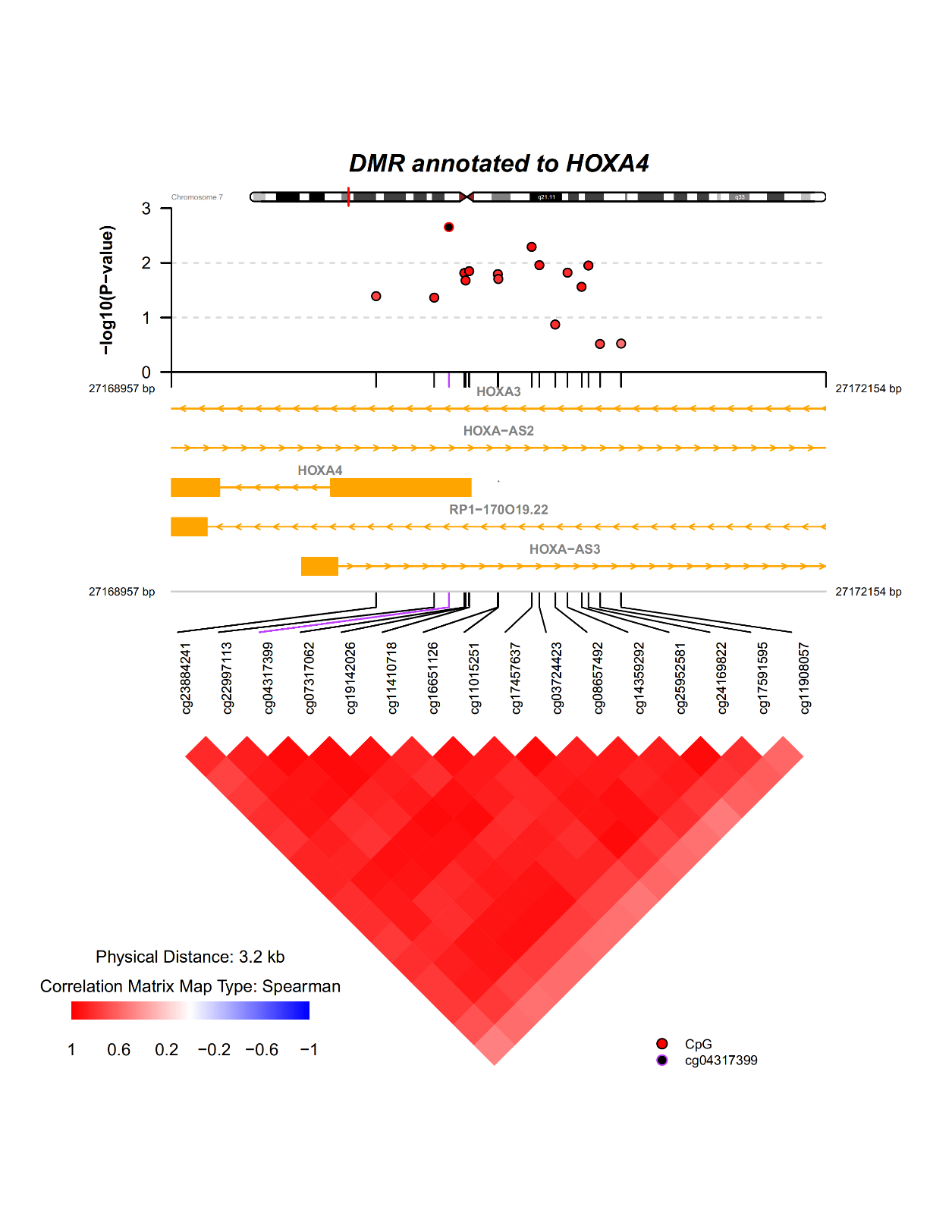
***

## **(8C)** The DMRs annotated to *HOXB6* (left) and *HOXB9* (right) were associated with the slope of CDR-SB.

**
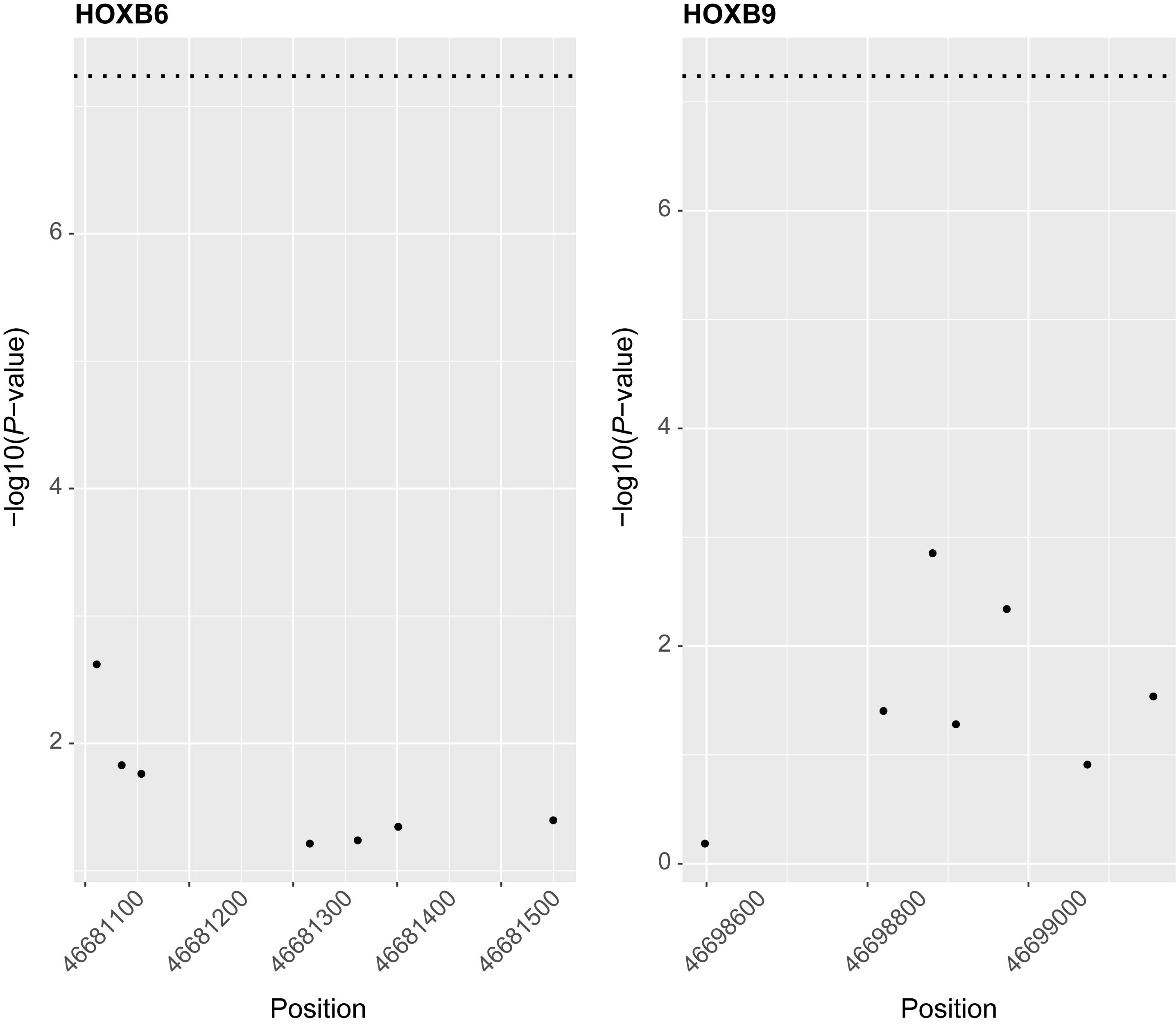
**

## **
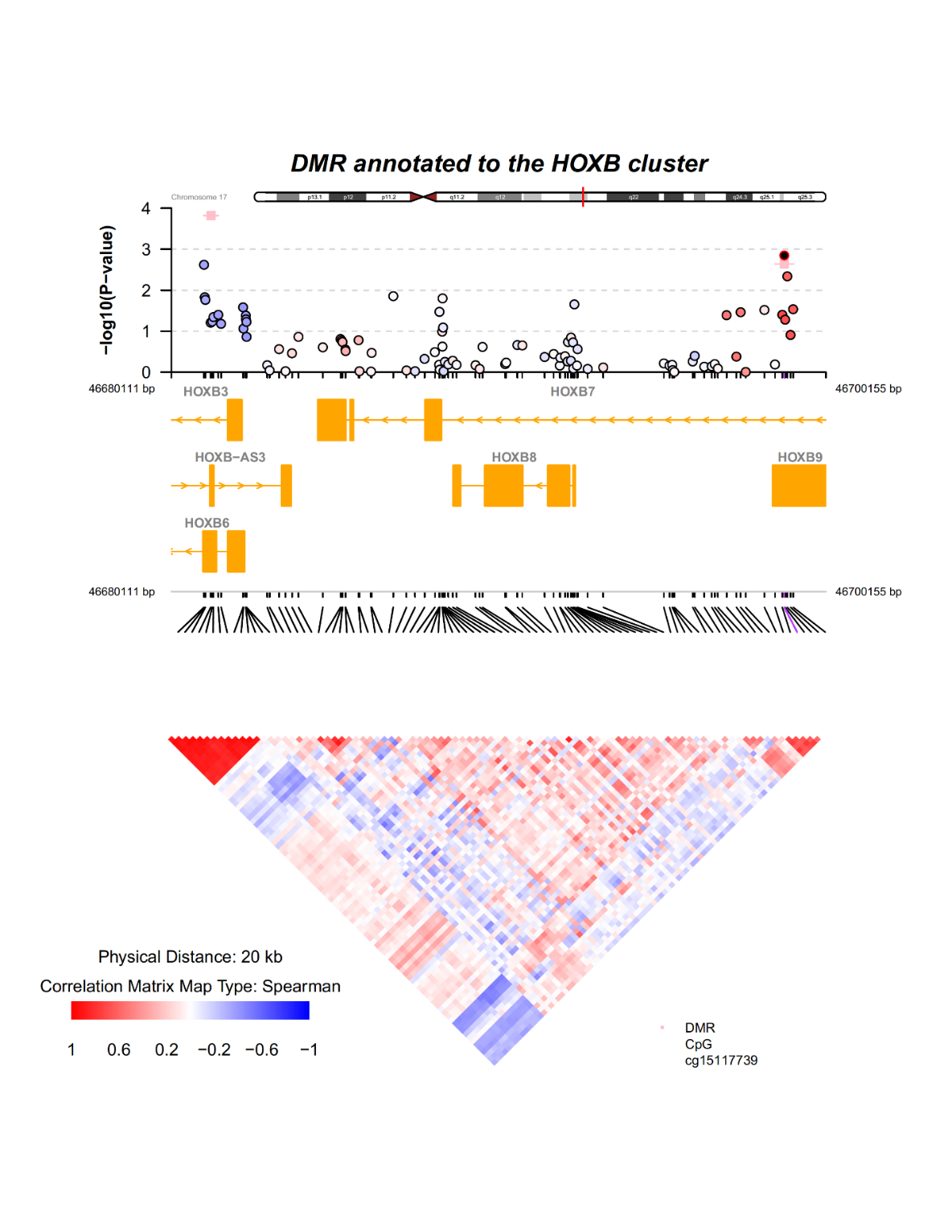
(8D)** Expanded *HOXB* gene cluster including the DMRs annotated to *HOXB6*/*HOXB9* (Figure 7C) were associated with the slope of CDR-SB. Pink filled squares denote DMRs and filled circles denote CpG sites.

## **(8E)** The DMRs annotated to *GABBR1* were associated with the MCI to AD conversion status.


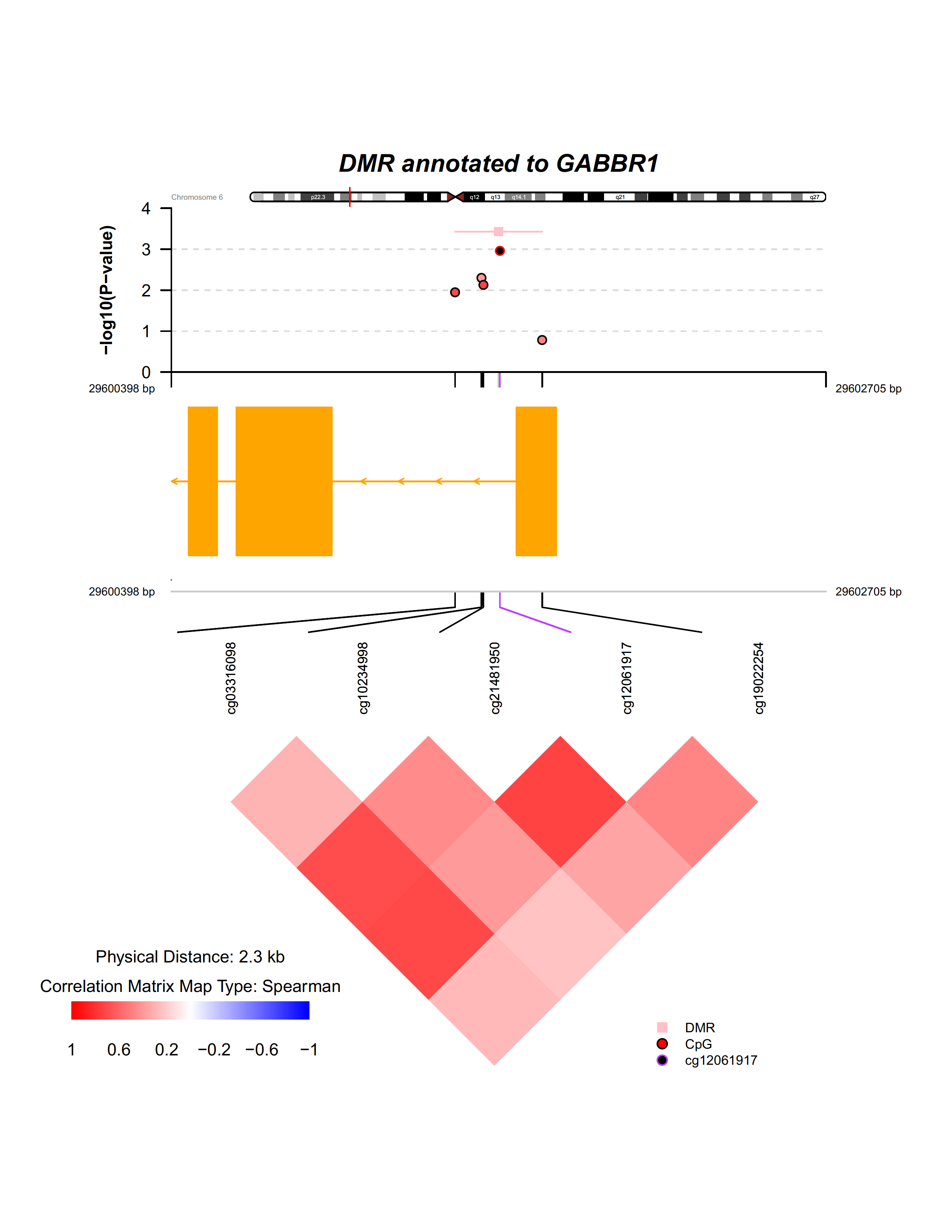


## **(8F)** The DMRs annotated to *DUSP22* were associated with the CN to MCI conversion status.

**
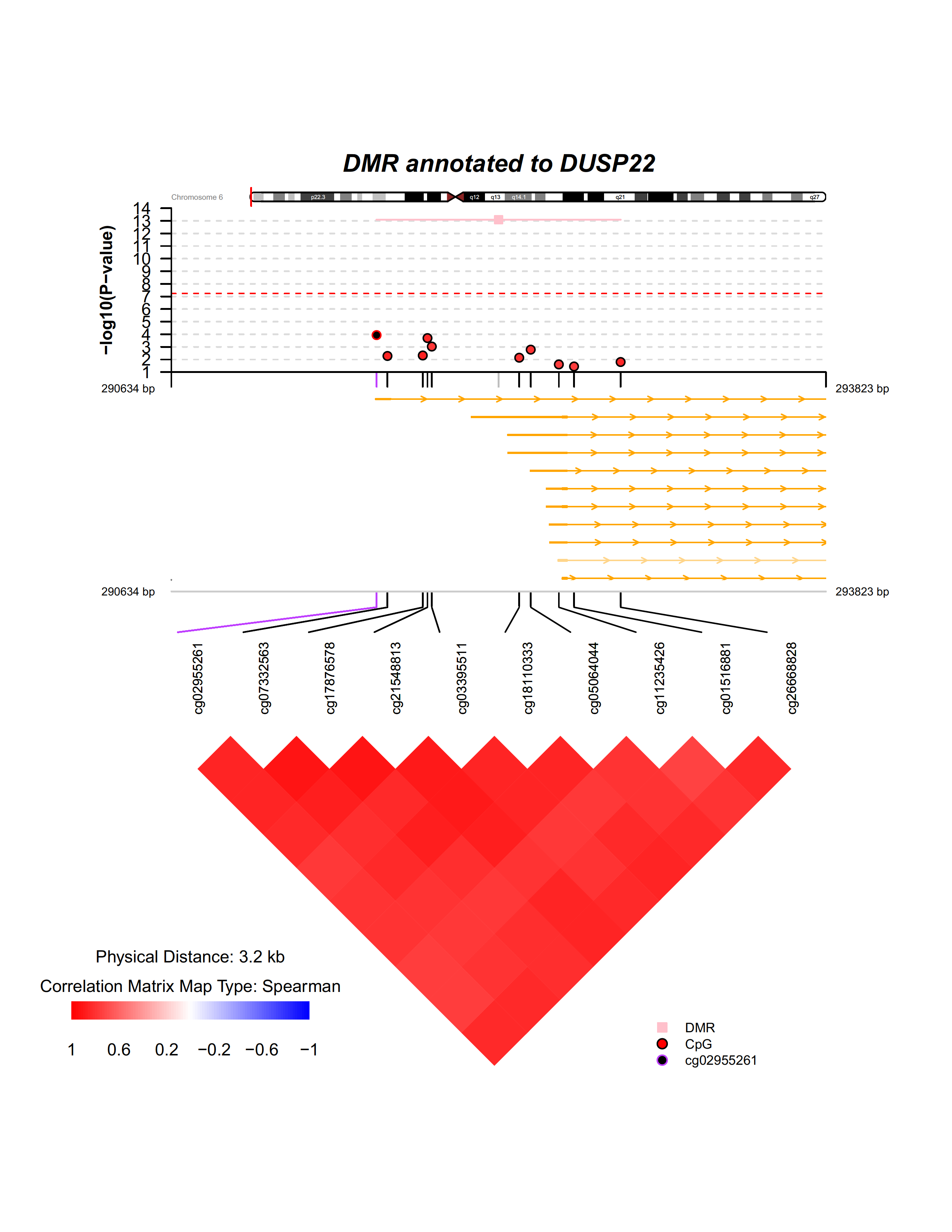
**

## **(8G)** The DMRs annotated to *LMTK3* were associated with the slope of CDR-SB.

**
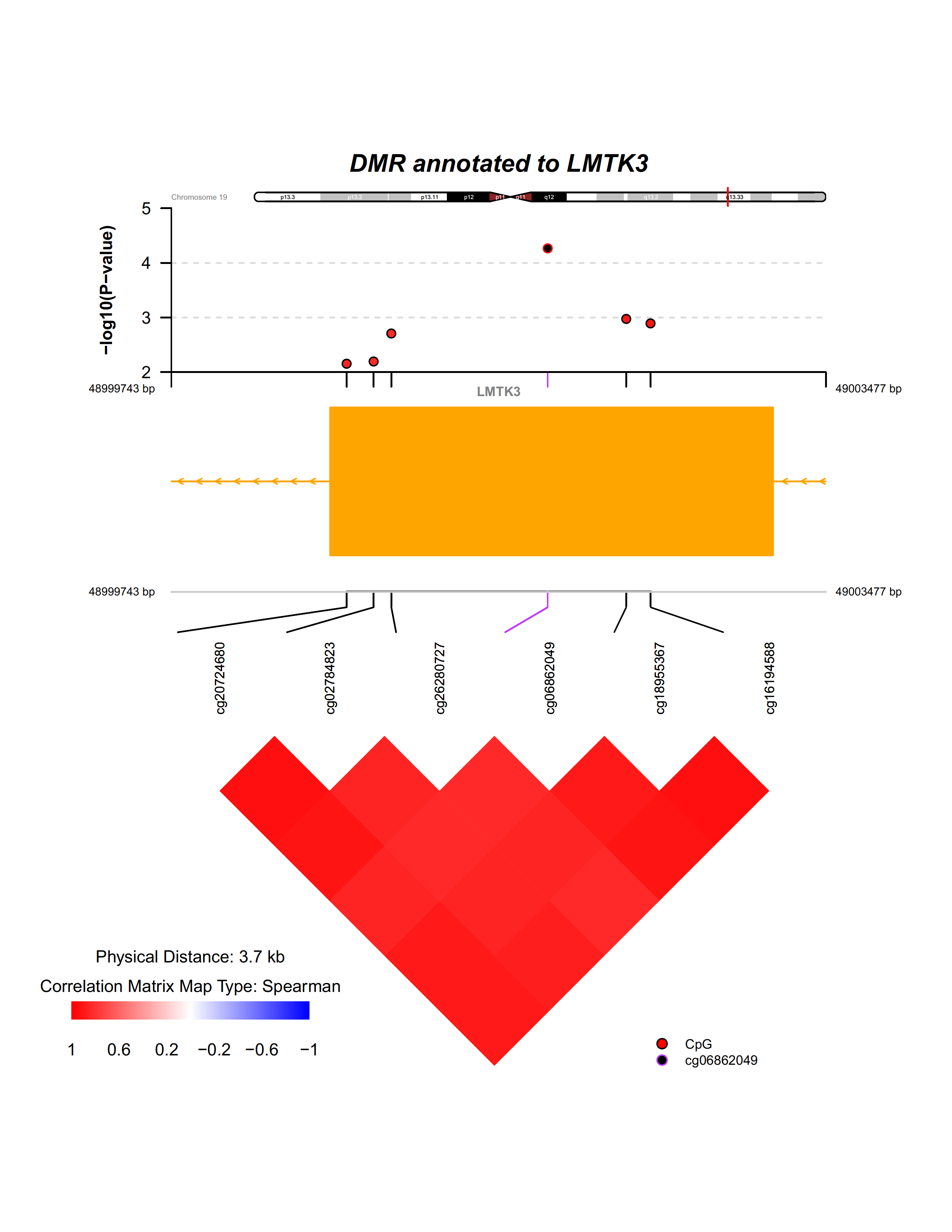
**

# **Supplemental Figure 9.** Cell type expression profile of (A) *ACY3* (B) *DUSP22* (C) *RPL37* (D) AMPD3 (E) *GABBR1* (F) *ATP6V0E2* (G) *NWD1* (H) *FGFR2.*

## (**9A**) ACY3.


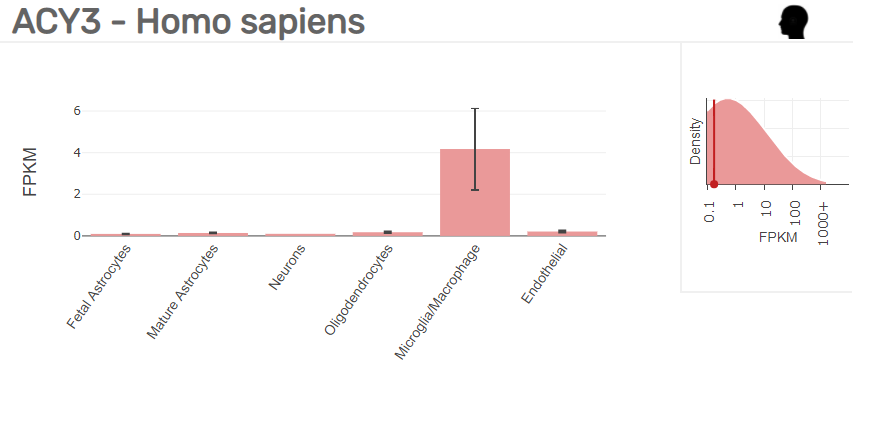


## (**9B**) DUSP22.


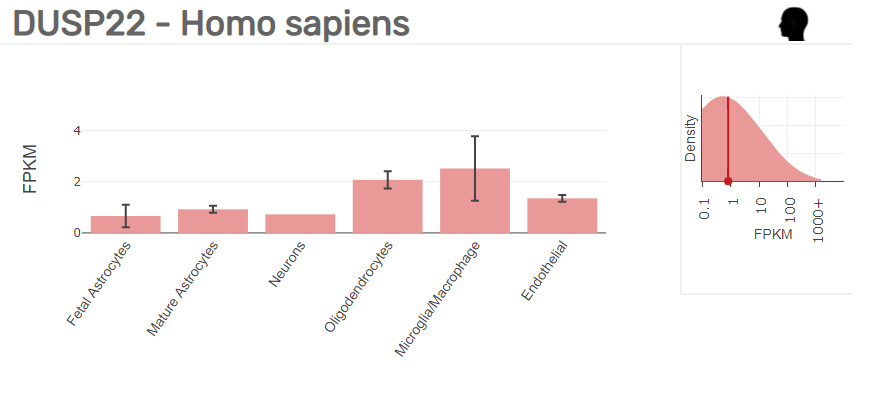


## (**9C**) RPL37.


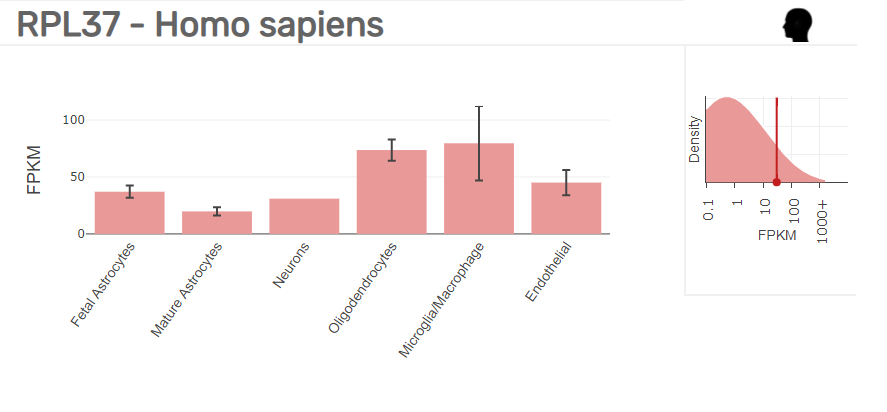


## (**9D**) AMPD3.


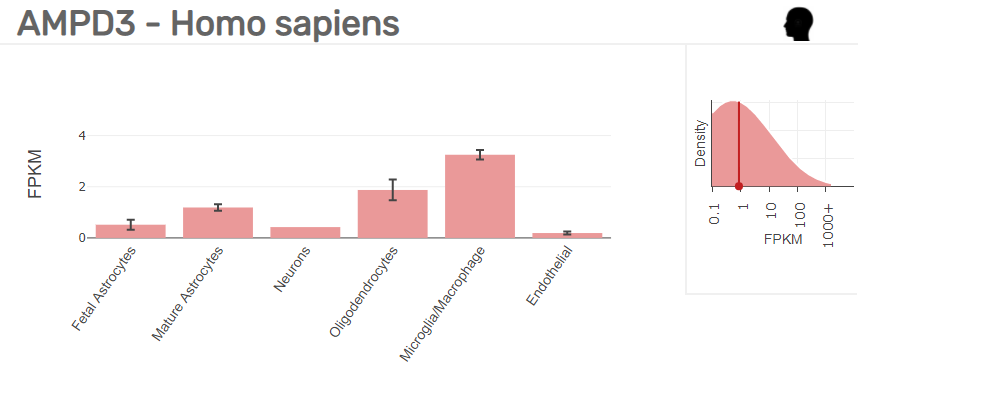


## (**9E**) GABBR1.


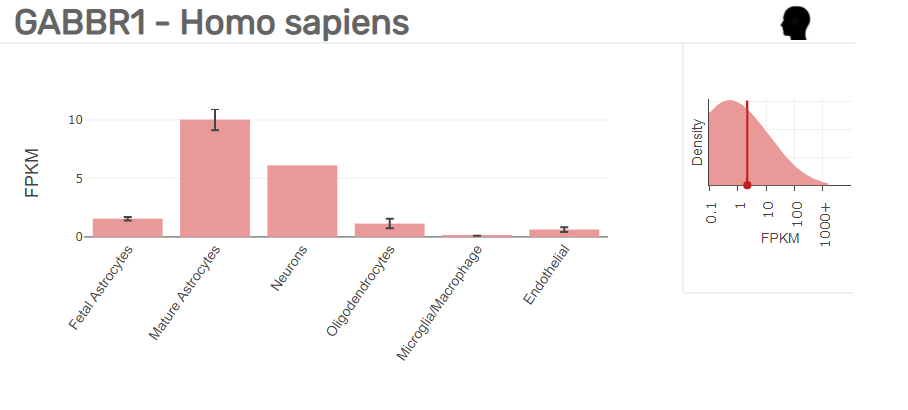


## (**9F**) ATP6V0E2.


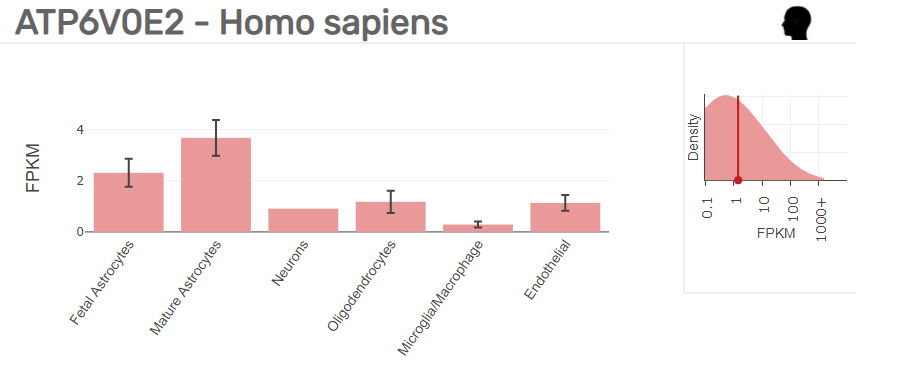


## (**9G**) NWD1.


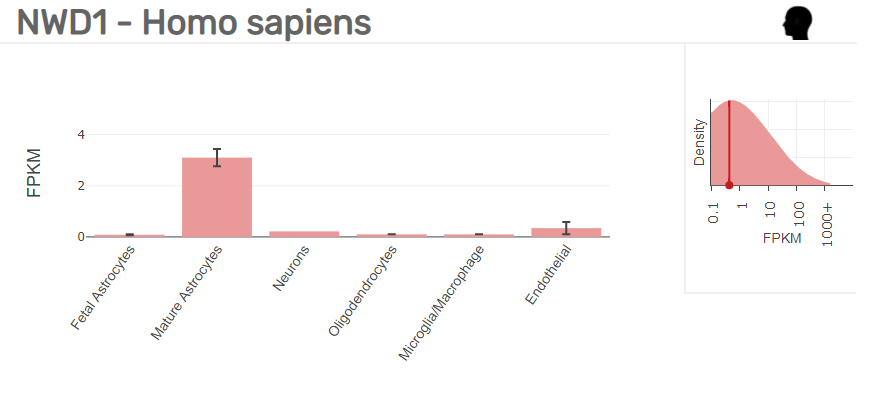


## (**9H**) FGFR2.


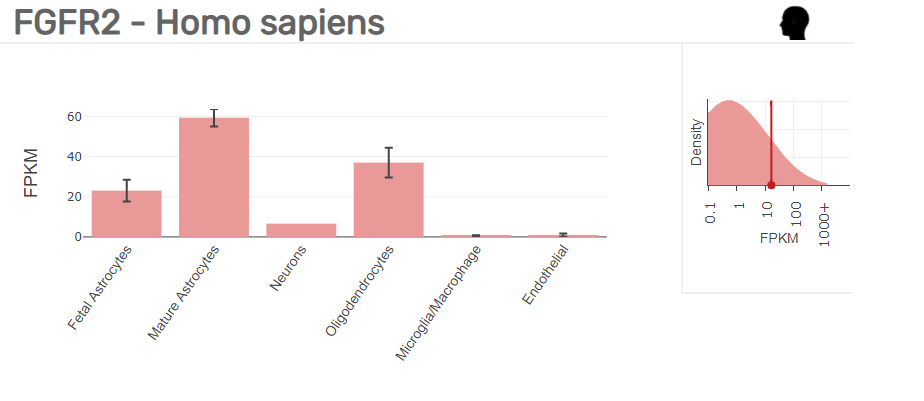


Source: https://www.brainrnaseq.org/

# **References**

1. Roubroeks JAY, Smith AR, Smith RG, Pishva E, Ibrahim Z, Sattlecker M, et al. An epigenome-wide association study of Alzheimer's disease blood highlights robust DNA hypermethylation in the HOXB6 gene. Neurobiol Aging. 2020;95:26-45.

2. Vasanthakumar A, Davis JW, Idler K, Waring JF, Asque E, Riley-Gillis B, et al. Harnessing peripheral DNA methylation differences in the Alzheimer's Disease Neuroimaging Initiative (ADNI) to reveal novel biomarkers of disease. Clin Epigenetics. 2020;12:84.

3. Smith RG, Pishva E, Shireby G, Smith AR, Roubroeks JAY, Hannon E, et al. A meta-analysis of epigenome-wide association studies in Alzheimer's disease highlights novel differentially methylated loci across cortex. Nat Commun. 2021;12:3517.
